# Optimal multiwave validation of secondary use data with outcome and exposure misclassification

**DOI:** 10.1002/cjs.11772

**Published:** 2023-03-31

**Authors:** Sarah C. LOTSPEICH, Gustavo G. C. AMORIM, Pamela A. SHAW, Ran TAO, Bryan E. SHEPHERD

**Affiliations:** 1 Department of Statistical Sciences, Wake Forest University, Winston-Salem, 27109, North Carolina, U.S.A.; 2 Department of Biostatistics, Vanderbilt University Medical Center, Nashville, 37203, Tennessee, U.S.A.; 3 Department of Biostatistics, Epidemiology, and Informatics, University of Pennsylvania, Philadelphia, 19104, Pennsylvania, U.S.A.; 4 Biostatistics Unit, Kaiser Permanente Washington Health Research Institute, Seattle, 98101, Washington, U.S.A.; 5 Vanderbilt Genetics Institute, Vanderbilt University Medical Center, Nashville, 37232, Tennessee, U.S.A.

**Keywords:** Data audit, HIV/AIDS, likelihood estimation, measurement error, two-phase design

## Abstract

Observational databases provide unprecedented opportunities for secondary use in biomedical research. However, these data can be error-prone and must be validated before use. It is usually unrealistic to validate the whole database because of resource constraints. A cost-effective alternative is a two-phase design that validates a subset of records enriched for information about a particular research question. We consider odds ratio estimation under differential outcome and exposure misclassification and propose optimal designs that minimize the variance of the maximum likelihood estimator. Our adaptive grid search algorithm can locate the optimal design in a computationally feasible manner. Because the optimal design relies on unknown parameters, we introduce a multiwave strategy to approximate the optimal design. We demonstrate the proposed design’s efficiency gains through simulations and two large observational studies.

## Résumé

Les bases de données observationnelles ouvrent des opportunités inédites pour la recherche biomédicale en raison de leur potentiel d’utilisation secondaire. Toutefois, les limites de ressources empêchent généralement la validation complète de ce type de base de données. Une méthode en deux phases qui vérifie un sous-ensemble d’enregistrements enrichis en informations pertinentes pour une question de recherche spécifique pourrait être utilisée. Les auteurs de cet article considèrent l’estimation du rapport de cotes en présence de biais de classification du résultat et de l’exposition ; ils proposent des plans optimaux qui minimisent la variance de l’estimateur du maximum de vraisemblance. Leur algorithme de recherche en grille adaptative est capable de trouver le plan optimal de manière efficace en termes de calcul. Sachant que ce plan optimal dépend de paramètres inconnus, ils utilisent une stratégie en plusieurs cycles de calcul pour son approximation. Enfin, ils mettent en évidence les gains d’efficacité du plan proposé grâce à des simulations et deux grandes études observationnelles.

## 1. INTRODUCTION

The ever-growing trove of patient information in observational databases, such as electronic health records (EHRs), provides unprecedented opportunities for biomedical researchers to investigate associations of scientific and clinical interest. However, these data are usually error-prone since they are “secondary use data,” i.e., they were not primarily created for research purposes (Safran et al., 2007). Ignoring these errors can yield biased results (Giganti et al., 2019), and the interpretation, dissemination, or implementation of such results can be detrimental to the very patients whom the analysis sought to help.

To assess the quality of secondary use data, validation studies have been carried out wherein trained auditors compare clinical source documents (e.g., paper medical records) to database values and note any discrepancies between them (Duda et al., 2012). The Vanderbilt Comprehensive Care Clinic (VCCC) is an outpatient facility in Nashville, Tennessee, that provides care for people living with HIV/AIDS (PLWH). Since investigators at the VCCC extract EHR data for research purposes, the VCCC validates all key study variables for all patients in the EHR. The VCCC data have demonstrated the importance of data validation, as estimates using the fully validated data often differ substantially from those using the original, unvalidated data extracted from the EHR (Giganti et al., 2020).

However, validating entire databases can be cost-prohibitive and unattainable; in the VCCC, full-database validation of around 4000 patients costs over US$60,000 annually. A cost-effective alternative is a two-phase design (White, 1982), or partial data audit, wherein one collects the original, error-prone data in Phase I and then uses this information to select a subset of records for validation in Phase II. This design greatly reduces the cost associated with data validation and has been implemented in cohorts using routinely collected data, such as the Caribbean, Central, and South America network for HIV Epidemiology (CCASAnet) (McGowan et al., 2007).

CCASAnet is a large (~50,000 patients), multinational HIV clinical research collaboration. Clinical sites in CCASAnet routinely collect important variables, and these site-level data are subsequently compiled into a collaborative CCASAnet database that is used for research. One interesting question for CCASAnet investigators is whether patients treated for tuberculosis (TB) are more likely to have better treatment outcomes if their TB diagnosis was bacteriologically confirmed. TB is difficult to diagnose and treat among PLWH, and some studies suggest that those treated for TB without a definitive diagnosis are more likely to subsequently die (Crabtree-Ramirez et al., 2019). Key study variables can be obtained from the CCASAnet database, but the outcome and exposure, successful treatment completion and bacteriological confirmation, respectively, can be misclassified in the database. For more than a decade, partial data audits have been performed to ensure the integrity of the CCASAnet research database (Duda et al., 2012; Giganti et al., 2019; Lotspeich et al., 2020), and plans are currently under way to validate these TB study variables on a subset of records in the near future. Site-stratified random sampling has been the most common selection mechanism thus far, including a 2009–2010 audit of the TB variables. Now, we are interested in developing optimal designs that select subjects who are most informative for our study of the association between bacteriological confirmation and treatment completion to answer this question with the best possible precision.

Statistical methods have been proposed to analyze data from two-phase studies like this and correct for binary outcome misclassification and exposure errors simultaneously. These methods can largely be grouped into likelihood-based or design-based estimators. The former includes the maximum likelihood estimator (MLE) (Tang et al., 2015) and the semiparametric maximum likelihood estimator (SMLE) (Lotspeich et al., 2022), where the latter includes the inverse probability weighted (IPW) estimator (Horvitz & Thompson, 1952) and the generalized raking/augmented IPW estimator (Deville, Särndal & Sautory, 1993; Robins, Rotnitzky & Zhao, 1994; Oh et al., 2021b). Likelihood-based estimators are fully efficient when all models (i.e., analysis and misclassification models) are correctly specified, while design-based estimators tend to be more robust since they make fewer distributional assumptions (i.e., do not require specification of a misclassification model). We focus on full-likelihood estimators because we have full-cohort information and because these estimators offer greater efficiency. Theoretical properties and empirical comparisons of these estimators have been discussed in detail before (e.g., McIsaac & Cook, 2014; Lotspeich et al., 2022). Thus, in this article, we focus on designs, rather than estimation, for two-phase studies.

Given the resource constraints imposed upon data audits, efficient designs that target the most informative patients are salient. Closed-form solutions exist for the optimal sampling proportions to minimize the variances for some design-based estimators under settings with outcome (e.g., Pepe, Reilly & Fleming, 1994) or exposure error alone (e.g., Reilly & Pepe, 1995; McIsaac & Cook, 2014; Chen & Lumley, 2020). Optimal designs for likelihood-based estimators have also been considered in the setting of exposure errors alone, although the variances of these estimators do not lend themselves to closed-form solutions unless additional assumptions are made (Breslow & Cain, 1988; Holcroft & Spiegelman, 1999; McIsaac & Cook, 2014; Tao, Zeng & Lin, 2020).

Still, optimal designs have yet to be developed for two-phase studies with a misclassified binary outcome *and* exposure error, as needed for the CCASAnet TB study. Existing designs for our setting are limited to case–control (CC*) or balanced case–control (BCC*) designs based on the unvalidated outcome and exposure (Breslow & Cain, 1988) (we use the * here to differentiate these designs, which are based on error-prone data, from their traditional counterparts). In fact, many of the designs proposed for expensive variables (a setting similar to measurement error) are just CC* or BCC* sampling or variations of them. These proposed designs target a particular prevalence for the variable of interest (Tan & Heagerty, 2022) or “balanced” numbers from each of the Phase I strata (White, 1982; Wang et al., 2017), respectively, to sample in Phase II. While these designs are practical and can offer efficiency gains over simple random sampling (SRS), they were not derived to be optimal for any specific target parameter. Our goal is to compute optimal designs for likelihood-based estimators in the unaddressed setting of binary outcome and exposure misclassification.

Regardless of the estimator, optimal designs share common challenges; in particular, they require the specification of unknown parameters. To overcome this, multiwave strategies have been proposed that estimate the unknown parameters with an internal pilot study and use this information to approximate the optimal design (McIsaac & Cook, 2015; Chen & Lumley, 2020; Han et al., 2020; Shepherd et al., 2022). Instead of selecting one Phase II subsample, multiwave designs allow iterative selection in two or more waves of Phase II. This way, each wave gains information from the previous waves. So far, multiwave designs have been used only to adapt optimal designs for design-based estimators. We focus on designing multiwave validation studies to improve the statistical efficiency of likelihood-based estimators under outcome and exposure misclassification.

Based on the asymptotic properties of the two-phase MLE for logistic regression, we derive the optimal validation study design to minimize the variance of the log odds ratio (OR) under differential outcome and exposure misclassification. In the absence of a closed-form solution, we devise an adaptive grid search algorithm that can locate the optimal design in a computationally feasible and numerically accurate manner. Because the optimal design requires the specification of unknown parameters at the outset and thus is unattainable without prior information, we introduce a multiwave sampling strategy to approximate the optimal design in practice. Through extensive simulations, the proposed optimal designs are compared to CC* and BCC* sampling. Notable gains in efficiency can be seen not only with the optimal design but also with the multiwave approximation. Using the VCCC data, we evaluate the efficiency of various designs validating different subsets of the EHR data and compare them with the fully validated, full-cohort analysis. Finally, we implement our approach to design the next round of CCASAnet audits.

## 2. METHODS

### 2.1. Model and Data

Consider a binary outcome Y, binary exposure X, and covariates Z that are assumed to be related through the logistic regression model $Pr(Y=1|X,Z)={\left[1+exp\left\{-\left(\beta_{0}+\beta X+\beta_{z}^{⊤}Z\right)\right\}\right]}^{-1}$. Instead of Y and X, error-prone measures $Y^{*}$ and $X^{*}$, respectively, are available in an observational database. Covariates Z are also available and error free. An audit sample of size n of the N subjects in the database (n<N) will have their data validated. Let $V_{i}=1$ if subject *i*, i∈{1,…,N}, is audited, and 0 otherwise. The joint distribution of a complete observation is

(1) $$\text{Pr}\left(V,Y^{*},X^{*},Y,X,Z\right)=\text{Pr}\left(V|Y^{*},X^{*},Z\right)\text{Pr}\left(Y^{*}|X^{*},Y,X,Z\right)\text{Pr}\left(X^{*}|Y,X,Z\right)\text{Pr}(Y|X,Z)\text{Pr}(X|Z)\text{Pr}(Z),$$

where $Pr\left(V|Y^{*},X^{*},Z\right)$ is the validation sampling probability; Pr(Y∣X,Z) is the logistic regression model of primary interest; $Pr\left(Y^{*}|X^{*},Y,X,Z\right)$ and $Pr\left(X^{*}|Y,X,Z\right)$ are the outcome and exposure misclassification mechanisms, respectively; Pr(X∣Z) is the conditional probability of X given Z; and Pr(Z) is the marginal density or mass function of Z. Sampling (i.e., *V*) is assumed to depend only on Phase I variables $\left(Y^{*},X^{*},Z\right)$, so (Y,X) are missing at random (MAR) for unaudited subjects.

Equation (1) captures the most general situation with complex differential misclassification in the outcome and exposure and without any assumptions of independence between variables, but it addresses other common settings as special cases. For classical scenarios of outcome or exposure misclassification alone, set $X^{*}=X$ or $Y^{*}=Y$, respectively. For non-differential misclassification, let $Pr\left(Y^{*}|X^{*},Y,X,Z\right)=Pr\left(Y^{*}|Y,Z\right)$ or $Pr\left(X^{*}|Y,X,Z\right)=Pr\left(X^{*}|X,Z\right)$ (Keogh et al., 2020). The model can be further customized if one has more specific knowledge, perhaps from a previous audit, scientific context, or the literature. For example, if the exposure and covariates are assumed to be independent, then Pr(X∣Z)=Pr(X). Importantly, these customizations do not affect the derivation of the optimal design that follows.

All observations $\left(V_{i},Y_{i}^{*},X_{i}^{*},Y_{i},X_{i},Z_{i}\right),i\in \{1,\ldots ,N\}$, are assumed to be i.i.d. following Equation (1). The necessary unknowns in Equation (1)—specifically, $Pr\left(Y^{*}|X^{*},Y,X,Z\right),Pr\left(X^{*}|Y,X,Z\right)$, and Pr(X∣Z) —are assumed to follow additional logistic regression models. Model parameters are denoted together by θ; since we focus on estimating β, all other nuisance parameters are denoted by η such that $\theta ={\left(\beta ,\eta^{⊤}\right)}^{⊤}$. Given that $\left(Y_{i},X_{i}\right)$ are incompletely observed, the observed-data log-likelihood for θ is

(2) $$\ell_{N}(\theta )=\sum_{i=1}^{N}V_{i}\text{ln}\left\{\text{Pr}\left(Y_{i}^{*}|X_{i}^{*},Y_{i},X_{i},Z_{i}\right)\text{Pr}\left(X_{i}^{*}|Y_{i},X_{i},Z_{i}\right)\text{Pr}\left(Y_{i}|X_{i},Z_{i}\right)\text{Pr}\left(X_{i}|Z_{i}\right)\right\}+\sum_{i=1}^{N}\left(1-V_{i}\right)\text{ln}\left\{\sum_{y=0}^{1}\sum_{x=0}^{1}\text{Pr}\left(Y_{i}^{*}|X_{i}^{*},y,x,Z_{i}\right)\text{Pr}\left(X_{i}^{*}|y,x,Z_{i}\right)\text{Pr}\left(y|x,Z_{i}\right)\text{Pr}\left(x|Z_{i}\right)\right\}.$$

The distribution of V can be omitted because the Phase II variables are MAR. In other words, because $Pr\left(V|Y^{*},X^{*},Z\right)$ is fully observed (in fact, fixed by design) and only scales $\ell_{N}(\theta )$ by a constant, omitting it from Equation (2) does not affect parameter estimation. The MLE $\hat{\theta }={\left(\overset{ˆ}{\beta },{\overset{ˆ}{\eta }}^{⊤}\right)}^{⊤}$ can be obtained by maximizing Equation (2) (Tang et al., 2015). Our optimal design will obtain the most efficient MLE for β, the conditional log OR for X on Y.

### 2.2. Optimal Design

Under standard asymptotic theory with N→∞ and n/N→Pr(V=1)>0, $\sqrt{N}(\overset{ˆ}{\theta }-\theta )⇝𝒩\left(0,ℐ(\theta {)}^{-1}\right)$, where ⇝ represents convergence in distribution and $𝒩\left(0,ℐ(\theta {)}^{-1}\right)$ is a multivariate normal distribution with mean **0** and variance equal to the inverse of the Fisher information matrix, ℐ(θ). Partition ℐ(θ) as

$$ℐ(\theta )=\left[\begin{array}{cc} ℐ(\beta ,\beta ) & ℐ{\left(\beta ,\eta \right)}^{⊤}\\ ℐ(\beta ,\eta ) & ℐ(\eta ,\eta ) \end{array}\right].$$

The optimal design aims to minimize $Var(\overset{ˆ}{\beta })$, which can be expressed as

(3) $$\text{Var}(\hat{\beta })=N^{-1}{\left\{ℐ(\beta ,\beta )-ℐ{\left(\beta ,\eta \right)}^{⊤}ℐ{\left(\eta ,\eta \right)}^{-1}ℐ(\beta ,\eta )\right\}}^{-1}.$$

The elements of ℐ(θ) are expectations taken with respect to the complete data, following from the joint distribution in Equation (1). Thus, they can be expressed as functions of the sampling probabilities

$$\pi_{y^{*}x^{*}Z}\equiv \text{Pr}\left(V=1|Y^{*}=y^{*},X^{*}=x^{*},Z=z\right)$$

and model parameters θ. That is, for elements $\theta_{j}$ and $\theta_{j\prime }$ of θ

(4) $$ℐ\left(\theta_{j},\theta_{j^{\prime }}\right)=\sum_{y^{*}=0}^{1}\sum_{x^{*}=0}^{1}\sum_{z}\pi_{y^{*}x^{*}z}\sum_{y=0}^{1}\sum_{x=0}^{1}S^{v}\left(\theta_{j};y^{*},x^{*},y,x,z\right)S^{v}\left(\theta_{j^{\prime }};y^{*},x^{*},y,x,z\right)\text{Pr}\left(y^{*},x^{*},y,x,z\right)+\sum_{y^{*}=0}^{1}\sum_{x^{*}=0}^{1}\sum_{z}\left(1-\pi_{y^{*}x^{*}z}\right)S^{\bar{v}}\left(\theta_{j};y^{*},x^{*},z\right)S^{\bar{v}}\left(\theta_{j^{\prime }};y^{*},x^{*},z\right)\sum_{y=0}^{1}\sum_{x=0}^{1}\text{Pr}\left(y^{*},x^{*},y,x,z\right),$$

where $S^{\nu }(\cdot )$ and $S^{\overset{‾}{v}}(\cdot )$ are the score functions of validated and unvalidated subjects, respectively, and $Pr\left(Y^{*},X^{*},Y,X,Z\right)$ is the joint distribution of the error-prone and error-free variables (see the Appendix for details). The sampling strata are defined by $Y^{*},X^{*}$, and Z.

Note that Equation (4) implicitly assumes that the covariates Z are discrete. As will be seen in our applications, this is sometimes the case (e.g., Z is study site) but certainly not always (e.g., Z is a continuous lab value). If the covariates are continuous or multidimensional with many categories, one will need to simplify them to create sampling strata. Specifically, the covariates may need to be discretized or have their dimensions reduced to make sampling feasible; such a strategy has been employed by others (e.g., Lawless, Kalbfleisch & Wild, 1999; Zhou et al., 2002; Han et al., 2020; Tao, Zeng & Lin, 2020). The resulting optimal design based on the simplified covariates may no longer be optimal for minimizing the variance based on the full, unsimplified covariates, but in most scenarios it should be a good approximation to the optimal design and better than classical designs like the BCC* design (Tao, Zeng & Lin, 2020). Moreover, the resulting discretized design should converge to the optimal one as the number of strata increases. In practice, there also exists a trade-off between the pursuit of optimality (with more strata) and ease of implementation (with fewer strata).

We see from Equations (3)–(4) that the optimal design corresponds to the $\left\{\pi_{y^{*}x^{*}z}\right\}$ that minimize $Var(\overset{ˆ}{\beta })$ under the constraint

(5) $$\sum_{y^{*}=0}^{1}\sum_{x^{*}=0}^{1}\sum_{z}\pi_{y^{*}x^{*}z}N_{y^{*}x^{*}z}\equiv \sum_{y^{*}=0}^{1}\sum_{x^{*}=0}^{1}\sum_{z}n_{y^{*}x^{*}z}=n,$$

where $N_{y^{*}x^{*}z}$ and $n_{y^{*}x^{*}z}$ are the sizes of the stratum ($Y_{i}^{*}=y^{*},X_{i}^{*}=x^{*},Z_{i}=z$) in Phase I and Phase II, respectively. Because $\left\{N_{y^{*}x^{*}z}\right\}$ is fixed, finding the optimal value of $\left\{\pi_{y^{*}x^{*}z}\right\}$ is equivalent to finding the optimal value of $\left\{n_{y^{*}x^{*}z}\right\}$. Unfortunately, this constrained optimization problem does not have a closed-form solution, so we devise an adaptive grid search algorithm to find the optimal value of $\left\{n_{y^{*}x^{*}z}\right\}$.

### 2.3. Adaptive Grid Search Algorithm

The challenge at hand is one of combinatorics: of all the candidate designs that satisfy the audit size constraint and are supported by the available Phase I data (i.e., the stratum sizes $\left\{N_{y^{*}x^{*}z}\right\}$), we need to find the one that minimizes $Var(\overset{ˆ}{\beta })$. To locate the optimal design, we develop an adaptive grid search algorithm. Specifically, a series of grids are constructed at iteratively finer scales and over more focused candidate design spaces to locate the optimal design. The adaptive nature of our algorithm is necessitated by the dimensional explosion of the grid as the Phase I and Phase II sample sizes and the number of sampling strata increase.

Let K denote the number of sampling strata and let m denote the minimum number that must be sampled in a stratum; m is needed to avoid degenerate optimal designs where one or more sampling strata are empty, which would render the estimation of the conditional distribution of Phase I given Phase II data impossible (Breslow & Cain, 1988). In the first iteration of the grid search, we form the grid $G^{\left(1\right)}$ with candidate designs comprised of stratum sizes $\left\{n_{y^{*}x^{*}z}^{\left(1\right)}\right\}$ that satisfy the constraint in Equation (5) and

(6) $$\text{min}\;\left(m,N_{y^{*}x^{*}z}\right)\leq {n_{y^{*}x^{*}z}}^{\left(1\right)}\leq \text{min}\;\left(n-Km+m,N_{y^{*}x^{*}z}\right),$$

i.e., candidate stratum sizes fall between the minimum m and the maximum n−Km+m after minimally allocating to all K strata (or the full stratum size $N_{y^{*}x^{*}z}$ if either of these is not possible). The number of subjects $n_{y^{*}x^{*}z}^{\left(1\right)}$ in each stratum varies by a fixed quantity $s^{\left(1\right)}$ between candidate designs. For example, we might consider sampling $n_{y^{*}x^{*}z}^{\left(1\right)}\in \{10,25,\ldots ,100\}$ subjects if $s^{\left(1\right)}=15,m=10$, and $N_{y^{*}x^{*}z}=100$. We then calculate $Var(\overset{ˆ}{\beta })$ under each candidate design to identify the best one, i.e., the one that yields the smallest $Var(\overset{ˆ}{\beta })$. Given the large space of legitimate designs in this initial search, we want to choose a reasonably large $s^{\left(1\right)}$ to keep the dimension of $G^{\left(1\right)}$ manageable. Clearly, starting with a large $s^{\left(1\right)}$ will lead to a rough estimate of the optimal design, but this will be refined in subsequent iterations.

In the tth iteration (t>1), we form the grid $G^{\left(t\right)}$ around the $s^{\left(t-1\right)}$-person “neighbourhood” of the best design from the (t−1)th iteration whose best stratum sizes are denoted by $\left\{n_{y^{*}x^{*}z}^{\left(t-1\right)}\right\}$. That is, we construct $G^{\left(t\right)}$ from candidate designs that satisfy the constraint in Equation (5) and

(7) $$\text{max}\;\left(n_{y^{*}x^{*}z}^{\left(t-1\right)}-s^{\left(t-1\right)},m\right)\leq {n_{y^{*}x^{*}z}}^{\left(t\right)}\leq \text{min}\;\left(n_{y^{*}x^{*}z}^{\left(t-1\right)}+s^{\left(t-1\right)},N_{y^{*}x^{*}z}\right).$$

This constraint is a refined version of (6) since we focus on a narrower space of designs surrounding the previous iteration’s best design. Once again, the stratum sizes $\left\{n_{y^{*}x^{*}z}^{\left(t\right)}\right\}$ vary by multiples of $s^{\left(t\right)}$ between candidate designs. We adaptively choose $s^{\left(t\right)}<s^{\left(t-1\right)}$ such that the grids in $\left\{G^{\left(t\right)}\right\}$ become finer and finer during the iterative process. The choice of the step sizes $s^{\left(1\right)},\ldots ,s^{\left(T\right)}$ will determine the computation time to complete the algorithm, but the grid search appears robust to these choices (Appendix S1 in the Supplementary Material). We stop at $s^{\left(T\right)}=1$, meaning that the final search was conducted at the one-person level, and the best design at the last iteration (T) is the optimal design, which we call the optMLE design.

**Figure 1:**
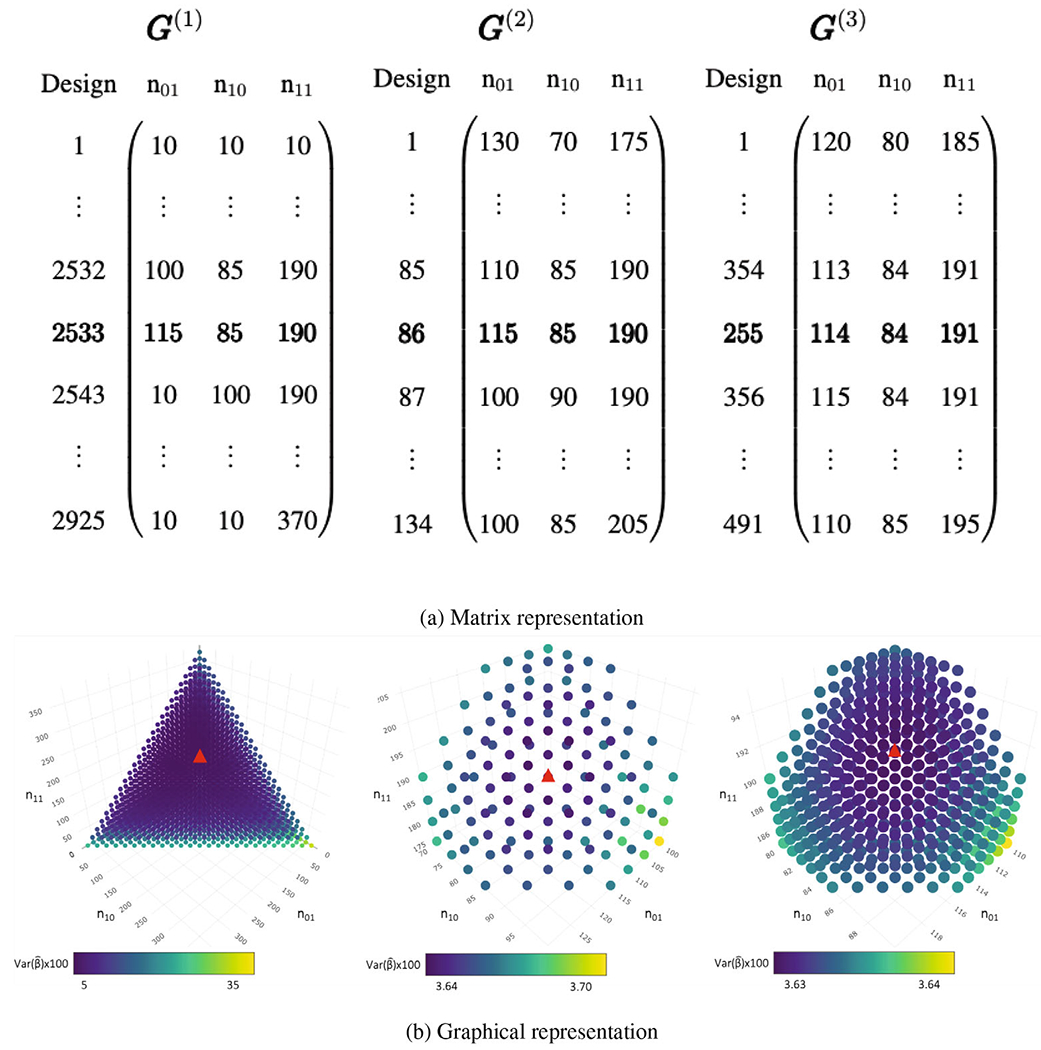
Matrix and graphical representations of a three-iteration adaptive grid search for a validation study of *n* = 400. In (a), the bold row indicates the design achieving the lowest $\text{Var}(\hat{\beta })$; in (b), the triangle indicates the same design. Stratum size *n*_00_ can be omitted because it is determined by the constraint in Equation (5).

Figure 1 depicts a schematic diagram of an adaptive grid search with T=3 iterations. In this hypothetical example, the Phase I sample size is N=10,000 and there are K=4 strata defined by $\left(Y^{*},X^{*}\right)$ with Phase I stratum sizes $\left(N_{00},N_{01},N_{10},N_{11}\right)=(5297,1130,2655,918)$. The aim is to select n=400 subjects for data validation in Phase II. Based on our simulations (discussed in Section 3.2), we set m=10. Assume that reliable parameter estimates are available from a previous data audit which can be used in the grid search. In the first iteration, we construct $G^{\left(1\right)}$ with candidate designs that satisfy the constraints in Equations (5) and (6), and vary the stratum sizes $\left\{n_{y^{*}x^{*}}\right\}$ by multiples of $s^{\left(1\right)}=15$ between designs. $Var(\overset{ˆ}{\beta })$ is minimized at $3.6283\times 10^{-4}$ under the candidate design with Phase II stratum sizes $\left(n_{00}^{\left(1\right)},n_{01}^{\left(1\right)},n_{10}^{\left(1\right)},n_{11}^{\left(1\right)}\right)=(10,115,85,190)$. In the second iteration, we form the grid $G^{\left(2\right)}$ from the 15-person neighbourhood around $\left\{n_{y^{*}x^{*}}^{\left(1\right)}\right\}$ such that the candidate designs satisfy the constraints in Equations (5) and (7), with stratum sizes varying by multiples of $s^{\left(2\right)}=5$ between designs. $Var(\overset{ˆ}{\beta })$ is minimized under the same design as in the first iteration, i.e., $\left\{n_{y^{*}x^{*}}^{\left(2\right)}\}=\{n_{y^{*}x^{*}}^{\left(1\right)}\right\}$. In the third and last iteration, we form the grid $G^{\left(3\right)}$ in the five-person neighbourhood around $\left\{n_{y^{*}x^{*}}^{\left(2\right)}\right\}$ such that the candidate designs satisfy the constraints in Equations (5) and (7) with stratum sizes varying by multiples of $s^{\left(3\right)}=1$ between designs. $Var(\overset{ˆ}{\beta })$ is minimized at $3.6281\times 10^{-4}$ by the Phase II sample sizes $\left\{n_{y^{*}x^{*}}^{\left(3\right)}\}=(11,114,84,191\right)$, which is the optMLE design. We note that the minimum variance barely changed between iterations in this toy example; the algorithm proceeds anyway because the stopping rule is defined as the most granular grid search (i.e., a one-person scale with $s^{\left(T\right)}=1$). In practice, one may use other sensible rules that permit early stops to make the algorithm more computationally efficient, e.g., stopping when the minimum variance from successive iterations changes by less than 1%.

### 2.4. Multiwave Approximate Optimal Design

The optMLE design derived in Section 2.2 relies on the model parameters θ, which are unknown at the study outset. If available, historical data from a previous audit could be used to estimate θ. Otherwise, it would be difficult to implement the optMLE design in practice, so we propose a multiwave sampling strategy to approximate it. Whereas traditional two-phase studies require all design-relevant information to be available in Phase I, multiwave designs allow sampling to adapt as such information accumulates through multiple sampling waves in Phase II.

In one of the earliest multiwave papers, McIsaac & Cook (2015) considered the most and least extreme multiwave sampling strategies: (i) fully adaptive and (ii) two waves, respectively. Strategy (i) begins with a small initial sample, after which the sampling strategy is recomputed after data are collected for each individual included in Phase II (leading to nearly n waves), while strategy (ii) uses just two waves where the study is redesigned just once (after the initial sample). McIsaac & Cook (2015) found that fully adaptive designs offer great efficiency, but that their implementation can be unrealistic in practice; meanwhile, the more practical two-wave strategy offers near-optimal efficiency. Therefore, in this article we primarily consider two waves of sampling, labelled as Phase II(a) and Phase II(b), with corresponding sample sizes denoted by $n^{\left(a\right)}$ and $n^{\left(b\right)}$, respectively, where $n^{\left(a\right)}+n^{\left(b\right)}=n$.

McIsaac & Cook (2015) also examined different $n^{\left(a\right)}:n^{\left(b\right)}$ ratios and found that the 1:1 ratio appeared to strike a good balance between (i) more accurate Phase II(a) estimation of θ with larger $n^{\left(a\right)}$ and (ii) more flexible design optimization in Phase II(b) with larger $n^{\left(b\right)}$. Based on this result, we select $n^{\left(a\right)}=n^{\left(b\right)}=n/2$ subjects in each wave. In Phase II(a), we typically select subjects through BCC* sampling; other existing designs could be used depending on available information (e.g., Oh et al., 2021a). We then use Phase I and Phase II(a) data to compute the preliminary MLE of the parameters, denoted ${\hat{\theta }}^{\left(a\right)}$, where the validation indicator is $V_{i}=1$ if subject *i*, i∈{1,…,N}, was sampled in Phase II(a), and 0 otherwise. Then, we use the grid search algorithm in Section 2.3 with ${\overset{ˆ}{\theta }}^{\left(a\right)}$ to determine the optimal allocation of the remaining subjects in Phase II(b). We call our two-wave, approximate optimal design the optMLE-2 design. Following both waves of validation, the final MLE $\hat{\theta }$ is obtained by combining data from Phases I, II(a), and II(b) and a redefined validation indicator, with $V_{i}=1$ if subject *i*, i∈{1,…,N}, was audited in either wave of the optMLE-2 design. Thus, ensuing inference is based on the n audited and the N−n unaudited subjects, as with a single wave of audits.

## 3. SIMULATIONS

Our objective with these simulation studies is three-fold: (i) to describe the construction of the optimal designs, since there is not a closed form; (ii) to demonstrate the efficiency gains of the optimal designs over existing designs; and (iii) to investigate the robustness of the proposed designs to model misspecification. This is explored through settings with varied rates of misclassification (Section 3.2), additional error-free information to incorporate (Section 3.4), model misspecification of the misclassification mechanisms at the design stage (Section 3.5), and either outcome or exposure misclassification alone (Section 3.6).

### 3.1. Validation Study Designs

We compare the performance of five two-phase validation study designs under differential outcome and exposure misclassification.

SRS: All subjects in Phase I have an equal probability of inclusion in Phase II.

CC*: Subjects are stratified on $Y^{*}$, and separate random samples of equal size are drawn from each stratum.

BCC*: Subjects are jointly stratified on $\left(Y^{*},X^{*}\right)$ or $\left(Y^{*},X^{*},Z\right)$, and separate random samples of equal size are drawn from each stratum.

optMLE: Subjects are jointly stratified on $\left(Y^{*},X^{*}\right)$ or $\left(Y^{*},X^{*},Z\right)$, and stratum sizes are determined by the adaptive grid search algorithm. This design is included as a “gold standard” as it requires knowledge of θ.

optMLE-2: Subjects are jointly stratified on $\left(Y^{*},X^{*}\right)$ or $\left(Y^{*},X^{*},Z\right)$. In Phase II(a), n/2 subjects are selected by BCC*. In Phase II(b), n/2 more subjects are selected by the adaptive grid search algorithm, with θ estimated using Phase I and II(a) data.

We compared the designs using two precision measures: (i) relative efficiency (RE), defined as the ratio of empirical variances of $\overset{ˆ}{\beta }$ in the final analysis, and (ii) relative interquartile range (RI), defined as the ratio of the widths of the empirical interquartile range (IQR) (McIsaac & Cook, 2015). The optMLE design based on the true parameter value θ and observed Phase I stratum sizes $\left\{N_{y^{*}x^{*}}\right\}$ or $\left\{N_{y^{*}x^{*}z}\right\}$ was treated as the reference design when calculating the RE and RI (i.e., the variance and IQR, respectively, of the optMLE design were used in the numerators of these measures). RE and RI values greater than 1 indicate better precision than the optMLE design, while values below 1 indicate worse precision. We also considered alternative versions of the referential optimal design (Table S1 in the Supplementary Material), but the results were similar to those for the optMLE design and thus are not included in subsequent simulations.

### 3.2. Outcome and Exposure Misclassification

We simulated data for a Phase I sample of N=10,000 subjects according to Equation (1). We generated X and Y from Bernoulli distributions with $p_{x}=Pr(X=1)$ and $Pr(Y=1|X)={\left[1+exp\left\{-\left(\beta_{0}+0.3X\right)\right\}\right]}^{-1}$. We used an approximate outcome prevalence of $p_{y0}=Pr(Y=1|X=0)$ to define $\beta_{0}=ln\left\{p_{y0}/\left(1-p_{y0}\right)\right\}$. We generated $Y^{*}$ and $X^{*}$ from Bernoulli distributions with $Pr\left(X^{*}=1|Y,X\right)={\left[1+exp\left\{-\left(\gamma_{0}+0.45Y+\gamma_{1}X\right)\right\}\right]}^{-1}$ and $Pr\left(Y^{*}=1|X^{*},Y,X\right)={\left[1+exp\left\{-\left(\alpha_{0}+0.275X^{*}+\alpha_{1}Y+0.275X\right)\right\}\right]}^{-1}$, where $\left(\gamma_{0},\gamma_{1}\right)$ and $\left(\alpha_{0},\alpha_{1}\right)$ control the strength of the relationship between error-prone and error-free variables. We define the “baseline” false positive and true positive rates for $X^{*}$, denoted by ${FPR}_{0}\left(X^{*}\right)$ and ${TPR}_{0}\left(X^{*}\right)$, respectively, as the false positive and true positive rates of $X^{*}$ when Y=0. Similarly, ${FPR}_{00}\left(Y^{*}\right)$ and ${TPR}_{00}\left(Y^{*}\right)$ are the baseline false positive and true positive rates for $Y^{*}$ when X=0 and $X^{*}=0$. With these definitions, we have

$$\gamma_{0}=-\text{ln}\;\left\{\frac{1-{\text{FPR}}_{0}\left(X^{\text{*}}\right)}{{\text{FPR}}_{0}\left(X^{\text{*}}\right)}\right\},\;\;\;\;\gamma_{1}=-\text{ln}\;\left\{\frac{1-{\text{TPR}}_{0}\left(X^{\text{*}}\right)}{{\text{TPR}}_{0}\left(X^{\text{*}}\right)}\right\}-\gamma_{0},$$

$$\alpha_{0}=-\text{ln}\;\left\{\frac{1-{\text{FPR}}_{00}\left(Y^{\text{*}}\right)}{{\text{FPR}}_{00}\left(Y^{\text{*}}\right)}\right\},\;\text{and}\;\;\alpha_{1}=-\text{ln}\;\left\{\frac{1-{\text{TPR}}_{00}\left(Y^{\text{*}}\right)}{{\text{TPR}}_{00}\left(Y^{\text{*}}\right)}\right\}-\alpha_{0}.$$

Both $Y^{*}$ and $X^{*}$ were misclassified, but the misclassification rates were varied separately; we fixed FPR(⋅)=0.1 and TPR(⋅)=0.9 for one variable and varied FPR(⋅)∈{0.1,0.5} and TPR(⋅)∈{0.9,0.5} for the other. We considered cases where Y=0 or 1 was more common by fixing $p_{x}=0.1$ and varying $p_{y0}$ from 0.1 to 0.9. Similarly, cases where X=0 or 1 was more common were considered by fixing $p_{y0}=0.3$ and varying $p_{x}$ from 0.1 to 0.9. The R code to replicate all simulations can be found at https://github.com/sarahlotspeich/auditDesignR.

Using the designs in Section 3.1, n=400 subjects were selected in Phase II. We considered minimum stratum sizes of m ranging from 10 to 50 for the optMLE design; all yielded stable estimates (Figure S1 in the Supplementary Material), so m=10 was used thereafter. In choosing m, there is a trade-off between the stability of the design and the potential efficiency gain, driven by larger and smaller choices of m, respectively. While the grid search parameters varied between replicates, three-iteration grid searches with step sizes s={15,5,1} and s={25,5,1} were most commonly used to locate the optMLE and optMLE-2 designs, respectively. Each setting was replicated 1000 times.

Tables 1 and 2 show that the optMLE-2 design was highly efficient, with RE >0.9 and RI >0.95 in most settings. In some settings, the RE and RI for the optMLE-2 design were even slightly larger than 1; this is because the optMLE design is asymptotically optimal but may not necessarily be optimal in finite-sample settings. In most settings, the optMLE-2 design exhibited sizeable efficiency gains over the BCC*, CC*, and SRS designs, with gains as high as with gains as high as 43%, 74%, and 83%, respectively. The efficiency gains were higher when the misclassification rates were lower, particularly for $Y^{*}$, or when Phase I stratum sizes were less balanced.

Even under the most severe misclassification, e.g., when ${FPR}_{00}\left(Y^{*}\right)$ and ${TPR}_{00}\left(Y^{*}\right)$ were both 0.5, the MLE should remain identifiable because of the validation sample. However, the CC* and SRS designs incurred bias—as much as 22% and 27%, respectively—primarily when $p_{y0}$ was further from 0.5; in these situations, the imbalance in Y made these designs susceptible to empty cells in the validation data. The optimal and BCC* designs were reasonably unbiased in all settings. However, we saw the smallest efficiency gain for the optimal designs when the misclassification rates were highest since the Phase I data were not very informative for Phase II.

The grid search successfully located the optMLE and optMLE-2 designs in all and ≥95% of replicates per setting, respectively. The grid search failed to locate the optMLE-2 design in a few replicates because empty cells in the cross-tabulation of unvalidated and validated data from the Phase II(a) sample, e.g., no false negatives for the exposure, led to unstable coefficients from logistic regression that rendered singular information matrices. This can happen when error rates are extreme in either direction. When error rates are extremely low, error-prone variables can become collinear with their error-free counterparts. In this case, we might treat the variable as error free and use the Phase I version of this variable in all models. When error rates are extremely high, we might not observe any cases of agreement in Phase II(a), e.g., when there are no records with $X^{*}=X$. In this case, more than two waves of Phase II might be needed to “fill in” the empty cells. Fortunately, we did not encounter this problem very often; out of 20,000 total replicates across these settings, we discarded 172 (0.9%).

Figure S2 in the Supplementary Material shows the average Phase II stratum sizes for all designs under the settings described. The make-up of the optMLE design depended on the Phase I stratum sizes and misclassification rates. It oversampled subjects from the less frequent strata. Furthermore, this oversampling was more extreme when the misclassification rates for the variable were higher. The optMLE-2 design had a similar but less extreme allocation compared to the optMLE design because the optMLE-2 design contained a BCC* sample of 200 subjects in Phase II(a). When the Phase I variables were not very informative about the Phase II variables, e.g., when ${FPR}_{0}\left(X^{*}\right)={TPR}_{0}\left(X^{*}\right)=0.5$, the optimal designs became less dependent on the Phase I stratum sizes, and the optMLE-2 design became less similar to the optMLE design because estimating θ was harder (Figure S3 in the Supplementary Material). With a larger minimum of m=50, allocations of the optMLE and optMLE-2 designs were more similar than with m=10, especially when the Phase I variables were informative (Figure S4 in the Supplementary Material).

### 3.3. More than Two Waves of Validation

We considered another approximately optimal design, the optMLE-3 design, which conducts validation in three waves. Phase II(a) was the same as in the optMLE-2 design, with n/2 subjects selected by BCC* sampling based on ($\left.Y^{*},X^{*}\right)$. Then, n/4 subjects were selected in Phases II(b) and II(c) by the adaptive grid search algorithm, with θ estimated from Phases I and II(a) and from Phases I, II(a), and II(b), respectively. Data were generated following Section 3.2 with $p_{x}=0.1,p_{y0}=0.3$, varied outcome misclassification rates, and fixed ${FPR}_{0}\left(X^{*}\right)=0.1$ and ${TPR}_{0}\left(X^{*}\right)=0.9$ for the exposure. Efficiency gains for optMLE-3 were similar to those for the optMLE-2 design, with both recovering 88% of the efficiency of the optMLE design (Table 3). The designs chose almost identical stratum sizes for validation (Figure S5 in the Supplementary Material). Other allocations of the Phase II sample across multiple validation waves are of course possible; we refer the reader to McIsaac & Cook (2015) for additional considerations.

### 3.4. Incorporating an Additional Error-free Covariate

We performed an additional set of simulations that incorporated an error-free covariate into the designs and analyses. Simulation details are in Appendix S2.1 in the Supplementary Material. In summary, the optMLE-2 design continued to be highly efficient, with gains as high as 43%,56%, and 59% over the BCC*, CC*, and SRS designs, respectively (Table S2 in the Supplementary Material). The optimal designs favoured sampling subjects from strata with larger Var(X∣Z=z), where the true value of X was harder to “guess” (Figure S5 in the Supplementary Material).

### 3.5. Optimal Designs’ Robustness to Model Misspecification

Next, we investigated the impact of model misspecification at the design stage on the efficiency of subsequent estimators. We simulated data using Equation (1) for a Phase I sample of N=10,000 subjects. We generated an additional error-free binary covariate Z along with X and Y from Bernoulli distributions with $Pr(Z=1)=0.25,Pr(X=1|Z)=[1+exp\{-(-2.2+0.5Z)\}{]}^{-1}$, and $Pr(Y=1|X,Z)=[1+exp\{-(-0.85+0.3X+0.25Z)\}{]}^{-1}$. We set ${FPR}_{0}\left(X^{*}\right)={FPR}_{00}\left(Y^{*}\right)=0.25$ and ${TPR}_{0}\left(X^{*}\right)={TPR}_{00}\left(Y^{*}\right)=0.75$, such that $X^{*}$ and $Y^{*}$ were generated from Bernoulli distributions with

$$\text{Pr}\left(X^{*}=1|Y,X,Z\right)={\left[1+\text{exp}\left\{-\left(-1.1+0.45Y+2.2X+Z+\delta_{1}XZ\right)\right\}\right]}^{-1}$$

and

$$\text{Pr}\left(Y^{*}=1|X^{*},Y,X,Z\right)={\left[1+\text{exp}\left\{-\left(-1.1+0.275X^{*}+2.2Y+0.275X+Z+\delta_{2}XZ\right)\right\}\right]}^{-1}$$

with $\delta_{1}$ and $\delta_{2}$ varied between −1 and 1.

We defined eight $\left(Y^{*},X^{*},Z\right)$ sampling strata and selected n=400 subjects in Phase II. Additional optimal designs, denoted optMLE* and optMLE-2*, assume only main effects for $Pr\left(X^{*}=1|Y,X,Z\right)$ and $Pr\left(Y^{*}=1|X^{*},Y,X,Z\right)$; clearly, these models will be misspecified at the design stage when $\delta_{1}$ or $\delta_{2}$ are nonzero. The analysis models were correctly specified (i.e., included the interaction term), although Lotspeich et al. (2022) found the MLE to be fairly robust to model misspecification in this setting.

Simulation results for the proposed designs can be found in Table 4. Even though the optMLE* and optMLE-2* designs were computed based on incorrect model specifications, very little efficiency was lost relative to the correctly specified gold-standard optMLE design. Moreover, the optMLE-2* design remained more efficient than existing designs, with efficiency gains as high as 47%, 43%, and 37% over the BCC*, CC*, and SRS designs, respectively (Table S3 in the Supplementary Material). Thus, the proposed optimal designs appeared to maintain their advantages even when we were uncertain about the model specification at the design stage. This includes the problematic situation where, at the design stage, we incorrectly omitted an interaction term from our misclassification model. The average validation sample sizes in each stratum for all designs are displayed in Figure S6 in the Supplementary Material. The differences between the optMLE and optMLE* designs, or between the optMLE-2 and optMLE-2* designs, were almost always small, although more visible when the model for $Y^{*}$ was misspecified.

### 3.6. Classical Scenarios with Outcome or Exposure Misclassification Alone

Detailed results for settings with outcome or exposure misclassification only are presented in Appendices S2.2 and S2.3, respectively, in the Supplementary Material. The optimal designs oversampled subjects from strata corresponding to the less frequent value of the error-prone variable (Figures S7 and S8 in the Supplementary Material). The optMLE-2 design approximated the optMLE design well and continued to offer sizable efficiency gains over existing designs (Table S4 in the Supplementary Material).

## 4. COMPARING PARTIAL- TO FULL-AUDIT RESULTS IN THE VCCC

The VCCC EHR contains routinely collected patient data, including demographics, antiretroviral therapy (ART), lab results (e.g., viral load and CD4 count), and clinical events. Since the VCCC data have been fully validated, available pre-/post-validation datasets can be used to compare two-phase designs and analyses that only validate a subset of records to the gold standard analysis that uses the fully validated data. We used these data to assess the relative odds of having an AIDS-defining event (ADE) within 1 year of ART initiation (Y) between patients who were/were not ART-naive at enrollment (X), while adjusting for square-root-transformed CD4 at ART initiation (Z). We extracted N=2012 records from the EHR for this study. In the unvalidated data $\left(Y^{*},X^{*}\right),73\%$ of patients were ART-naive at enrollment and 8% of patients experienced an ADE within 1 year.

The misclassification rate of ADE was 6%, with a 63% false positive rate (FPR) and only a 1% false negative rate (FNR). The misclassification rate of ART-naive status at enrollment was 11%, with FPR = 13% and FNR = 3%. Only 19 subjects (1%) had both outcome and exposure misclassification. CD4 count was error-free. We assumed misclassification models for ADE and ART status, with

$$\underset{\alpha }{\text{Pr}}(Y^{*}=1|X^{*},Y,X,Z)={\left[1+\text{exp}\left\{-\left(\alpha_{0}+\alpha_{1}X^{*}+\alpha_{2}Y+\alpha_{3}X+\alpha_{4}Z\right)\right\}\right]}^{-1}$$

and

$$\underset{\gamma }{\text{Pr}}(X^{*}=1|Y,X,Z)={\left[1+\text{exp}\left\{-\left(\gamma_{0}+\gamma_{1}Y+\gamma_{2}X+\gamma_{3}Z\right)\right\}\right]}^{-1},$$

respectively.

We defined four sampling strata according to unvalidated Phase I ADE and ART-naive status with stratum sizes $\left(N_{00},N_{01},N_{10},N_{11}\right)=(504,1350,42,116)$, where the first and second subscripts index error-prone ADE and ART naive status, respectively. We set n=200 and considered the optMLE-2, BCC*, CC*, and SRS designs. When implementing the optMLE-2 design, we selected $n^{\left(a\right)}=100$ subjects in Phase II(a) via BCC* sampling. All results were averaged over 1000 replicates except for the SRS and optMLE-2 designs. The SRS design encountered 118 replicates where the MLE was unstable or did not converge because of the very small numbers of audited events or exposures, while the grid search algorithm failed to locate the optMLE-2 design in 40 of the replicates. These problematic replicates were excluded. On average, the SRS, CC*, BCC*, and optMLE-2 audits chose $\left(n_{00},n_{01},n_{10},n_{11}\right)=(56,134,4,12),(27,73,26,74),(53,53,42,52)$, and (25, 39, 42, 95), respectively, from the four strata in Phase II.

Table 5 shows the results under the two-phase designs, plus those from the gold-standard and naive analyses using the fully validated and unvalidated data, respectively, from the full cohort. The log OR estimates under all two-phase designs were reasonably close to the gold-standard estimates and led to the same clinical interpretations: after controlling for $\sqrt{CD4}$ at ART initiation, ART-naive status at enrollment was not associated with changes in the odds of ADE within 1 year of ART initiation. The variance under the optMLE-2 design was 14%,13%, and 86% smaller than those under the BCC*, CC*, and SRS designs, respectively.

We also considered designs that further stratified on CD4 count. Specifically, we dichotomized CD4 count at its median, 238 cells/mm^3^, and formed strata defined by error-prone ADE, ART status, and CD4 category. The Phase I stratum sizes were $\left(N_{000},N_{010},N_{100},N_{110},\left.N_{001},N_{011},N_{101},N_{111}\right)=(171,701,34,93,333,649,8,23\right)$, where the third subscript indexes CD4 category, and 0 and 1 correspond to low and high CD4 counts, respectively. CD4 count was still treated continuously in all analyses. The SRS and CC* designs were unchanged because they do not incorporate exposure or covariate information. The BCC* design selected (28, 28, 28, 29, 28, 28, 8, 23) subjects in Phase II. The grid search algorithm successfully located the optMLE-2 design in 952 of the replicates (95%) with average Phase II stratum sizes of (14, 32, 33, 70, 13, 13, 8, 16). This resulted in a very minor variance reduction over the previous optMLE-2 design, which did not stratify by CD4 category (Table 5).

## 5. PROSPECTIVE AUDIT PLANNING IN CCASANET

Researchers are interested in assessing the association between bacteriological confirmation of TB and successful treatment outcomes among PLWH who are treated for TB. We are in the process of designing a multisite audit of n=500 patients to validate key variables and better estimate this association in the CCASAnet cohort. The outcome of interest (Y) is successful completion of TB treatment within 1 year of diagnosis; among patients who did not complete treatment, this captures unfavourable outcomes of death, TB recurrence, or loss to follow-up (with each of these outcomes also of interest in secondary analyses). The exposure of interest (X) is bacterial confirmation of TB, defined as any positive diagnostic test result, e.g., culture, smear, or PCR.

The Phase I sample comes from the current CCASAnet research database and includes all patients initiating TB treatment between 1 January 2010 and 31 December 2018. Error-prone values $\left(Y^{*},X^{*}\right)$ of the study variables are available on N=3478TB cases across sites in five countries (anonymously labelled as countries A–E) during this period. Patients were stratified on $\left(Y^{*},X^{*}\right)$ within countries A–E to create strata with ($N_{00},N_{01},N_{10},N_{11}$) equal to (704,246, 1015,415), (239,139, 336,218), (3, 7, 5, 17), (6, 9, 15, 14), and (12, 16, 36, 26), respectively, where the first and second subscripts index error-prone treatment completion and bacteriological confirmation, respectively.

To implement the optMLE-2 design as in Sections 3–4, $,n^{\left(a\right)}=250$ patients would be chosen in Phase II(a) using BCC* sampling from the $20\left(Y^{*},X^{*}\right.$, Country) strata. Alternatively, we could incorporate prior data from on-site chart reviews conducted in the five CCASAnet sites between 2009 and 2010. The original data from this time period captured a total of 595 TB cases (Phase I). In this historical dataset, 70% of cases completed treatment within 1 year and 68% had bacteriological confirmation of TB. Validated TB treatment and diagnosis were available for 40 subjects who were chosen for validation via site-stratified SRS. We observed 13% and 20% misclassification in $Y^{*}(FPR=7\%,FNR=23\%)$ and $X^{*}(FPR=39\%,FNR=5\%)$, respectively. No subject had both their outcome and exposure misclassified.

We demonstrate two ways to use these historical audits to design a more efficient validation study for the next round of CCASAnet audits. Strategy (i) estimates the parameters as ${\hat{\theta }}^{\left(h\right)}$ with the historical data, and uses ${\overset{ˆ}{\theta }}^{\left(h\right)}$ to derive the optMLE design to allocate n=500 subjects in one Phase II subsample. Strategy (ii) is a multiwave strategy that uses ${\hat{\theta }}^{\left(h\right)}$ to design Phase II(a) and then uses the Phase II(a) parameters, denoted ${\hat{\theta }}^{\left(a\right)}$, to design Phase II(b).

Given the small size of the historical audit (n=40), it was not possible to obtain country-level estimates of all parameters needed to derive the optimal design. Instead, we created country groupings (Z) based on the site-specific audit results (Table S5 in the Supplementary Material), where Z=0 for countries A and B with errors in $Y^{*}$ or $X^{*},Z=1$ for countries C and D with errors in both $Y^{*}$ and $X^{*}$, and Z=2 for country E with no errors. We assumed misclassification models for TB treatment completion and bacteriological confirmation with

$$\underset{\alpha }{\text{Pr}}(Y^{*}=1|X^{*},Y,X,Z)={\left(1+\exp\left[-\left\{\alpha_{0}+\alpha_{1}X^{*}+\alpha_{2}Y+\alpha_{3}X+\alpha_{4}\text{I}\left(Z=1\right)+\alpha_{5}\text{I}\left(Z=2\right)\right\}\right]\right)}^{-1}$$

and

$$\underset{\gamma }{\text{Pr}}(X^{*}=1|Y,X,Z)={\left(1+\text{exp}\left[-\left\{\gamma_{0}+\gamma_{1}Y+\gamma_{2}X+\gamma_{3}\text{I}(Z=1)+\gamma_{4}\text{I}(Z=2)\right\}\right]\right)}^{-1}.$$

These groupings were used to obtain the MLE ${\hat{\theta }}^{\left(h\right)}$ for the historic data. Since audits will be conducted at the site level, we applied these parameters to the 20 Phase I strata from the current data by assuming the same coefficients for countries in the same Z group (Table S6 in the Supplementary Material).

First, we derived the optMLE design for n=500 with m=10 using the historic audits (Strategy (i)), which was made up of $\left(Y^{*},X^{*}\right)$ strata from countries A–E with $\left(n_{00},n_{01},n_{10},n_{11}\right)$ equal to (10, 15, 10, 21), (20, 80, 11,168), (3, 7, 5, 17), (6, 9, 15, 14), and (12, 16, 35, 26), respectively. All or nearly all available subjects were taken from countries C–E. In countries A and B, subjects with $X^{*}=1$ were preferred, particularly when paired with $Y^{*}=1$. With fewer records from country B than A, the optMLE design selected more subjects from the former.

Then, we implemented the grid search to select $n^{\left(a\right)}=250$ subjects as a more informed first wave for the two-wave design (Strategy (ii)). Based on the historical parameters, the stratum sizes to be sampled at Phase II(a) were $\left(n_{00}^{\left(a\right)},n_{01}^{\left(a\right)},n_{10}^{\left(a\right)},n_{11}^{\left(a\right)}\right)$, equal to (10, 10, 10, 10), (10, 10, 10, 45), (3, 7, 5, 17), (6, 9, 10, 13), and (12, 16, 11, 26) for countries A–E, respectively. Validation appeared focused on the smaller countries (C–E). Country A was sampled minimally, proportional to its Phase I sample size, as were all strata in country B except $\left(Y^{*}=1,X^{*}=1\right)$. Validated data on these subjects will be used to re-estimate the model parameters and derive the optimal allocation for Phase II(b). Alternatively, the Phase II(a) and historical data could be pooled to re-estimate the parameters. In our situation, the historical audits were much smaller than the Phase II(a) study, so Phase II(a) would likely dominate the pooled analysis. However, if the Phase II(a) study were smaller, e.g., due to budget constraints, then the benefits of data pooling could be greater.

Ultimately, the choice between these strategies is determined by logistics and our confidence in the historical data. We plan to use Strategy (ii): the optMLE-2 design with the first wave informed by prior audits. Incorporating the prior audit information, even though it might be biased, will likely be better than starting off with a BCC* design (Chen & Lumley, 2020), but we do not want to trust the historical audits entirely. Also, conducting multiple validation waves is feasible because they can be performed by in-country investigators (Lotspeich et al., 2020).

## 6. DISCUSSION

**Figure 2:**
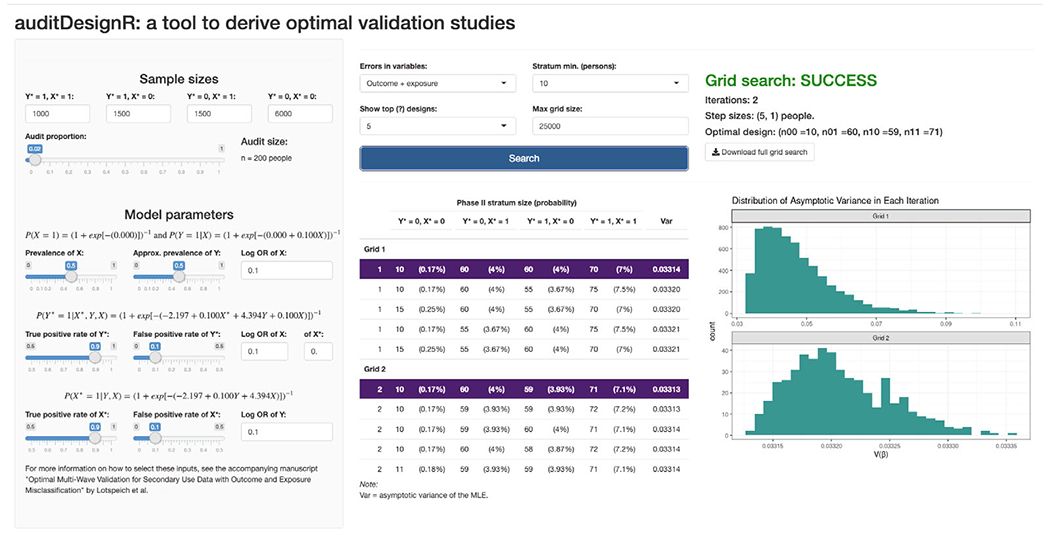
A screenshot of the auditDesignR Shiny application after a two-iteration adaptive grid search to find the optimal design. The user selects options from the sidebar (e.g., Phase I strata sizes and model parameters), followed by design-specific options such as the error setting to assume (e.g., errors in both the outcome and exposure) and the minimum required stratum size *m*. There are also controls for the adaptive grid search routine, like the maximum allowable grid size (here assumed to be 25,000 candidate designs). After making selections and clicking “Search,” the user can view the top candidate designs in addition to the distribution of $\text{Var}(\hat{\beta })$ from each iteration of the grid search.

Validation studies are integral to many statistical methods to correct for errors in secondary use data. However, they are resource-intensive undertakings. The numbers of records and variables that can be reviewed are limited by time, budget, and staffing constraints. Thus, selecting the most informative records is key to maximizing the benefits of validation. We introduced a new optimal design and a multiwave approximation that maximize the efficiency of the MLE under differential outcome and exposure misclassification—a setting for which optimal designs have not yet been proposed. We devised a novel adaptive grid search algorithm to locate the optimal design, which was implemented in the auditDesignR R package and the Shiny app (Figure 2) available in the Supplementary Material.

As part of our audit protocol, the CCASAnet sites are notified in advance with the list of patient records to be validated. This provides time for site investigators to locate the relevant patient charts before the audit, but there is still a chance that validation data may be missing. Our methods rely on the MAR assumption, which asserts that, conditioning on the Phase I information, subjects who are unaudited are similar to those who are audited. When we select who to audit, MAR holds because the validation data are missing by design, but validation data missing simply because we cannot find them calls the MAR assumption into question. In our previous CCASAnet audits, there have been instances where validation data are missing for selected records, although it is not very common. In the past, we have implicitly assumed that these records are MAR. Methods to simultaneously address audit data that are missing by design (i.e., MAR) and nonresponse (i.e., possibly not MAR) would be an interesting direction for future work.

Our analyses and designs are based on the parametric MLE approach of Tang et al. (2015). Recently, an SMLE approach was developed to analyze two-phase studies with error-prone outcome and exposure data that nonparametrically models the exposure-error mechanism, making it robust to different exposure-error mechanisms (Lotspeich et al., 2022). Our designs are guaranteed to be optimal for the MLE but still offer efficiency gains for the SMLE; this avoids complicated calculations that would otherwise be required to derive a design specifically for the SMLE. In an additional simulation, we found that the efficiency gains of the SMLE and MLE under the proposed optimal designs were essentially identical (Table S7 in the Supplementary Material).

We focused on designs for full-likelihood estimators because they offer the greatest efficiency if full-cohort information (i.e., data from both Phases I and II) is available, which was the case with the VCCC and CCASAnet examples. However, if only audit data are available or one wants to avoid placing additional models on the misclassification mechanisms, conditional maximum likelihood estimation (CMLE) could be considered (Breslow & Cain, 1988). Optimal designs for CMLE could be calculated in a similar manner, but we have not done so here.

Strictly speaking, the proposed optimal design is “optimal” among designs with compatible strata definitions only. In the VCCC example, we considered an optimal design that sampled on a categorical version of continuous CD4 count in addition to the error-prone outcome and exposure; we created two categories based on CD4 counts above or below the median. While we treated CD4 as discrete to compute the optimal design, performance with continuous CD4 in the analysis was still good. There are other ways we might have discretized CD4, e.g., by creating more than two categories or choosing a different cut-point than the median. How to best stratify continuous variables for design purposes is an interesting question (e.g., Amorim et al., 2021), and designs that maintain the continuous nature of continuous covariates warrant further investigation.

Other interesting topics for future research include developing optimal designs for two-phase validation studies with other types of outcomes and exposures, including count, continuous, or censored data. Developing these designs would involve replacing the models in Equations (1)–(4) with models appropriate for the new data types and then deriving successive steps in parallel to the current work. The special case with Phase I data made up of multiple error-prone surrogates for Y or X, as in a reliability study, would also be a natural extension of the methods herein. Also, the proposed designs could be modified to pursue optimal estimation of the interaction between the misclassified exposure and an additional covariate, similar to the focus of Breslow & Chatterjee (1999), or of multiple parameters simultaneously, e.g., the main effects and interaction. Either of these modifications would require the adoption of an alternative criterion to summarize the variance matrix, such as D-optimality or A-optimality, which minimizes the determinant or trace, respectively, of the variance matrix (Fedorov & Leonov, 2013).

## Supplementary Material

Supplemental Material

## Figures and Tables

**Table 1:** Simulation results under outcome and exposure misclassification with varied outcome misclassification rates.

Outcome misclassification		optMLE-2				BCC*				CC*				SRS			
FPR*_00_*(Y*)	TPR*_00_*(Y*)	% Bias	SE	RE	RI	% Bias	SE	RE	RI	% Bias	SE	RE	RI	% Bias	SE	RE	RI
*p*_*y*0_ = 0.1																	
0.1	0.9	−2.85	0.214	1.028	1.015	−4.00	0.254	0.728	0.855	−9.20	0.347	0.391	0.640	−19.4	0.516	0.176	0.459
	0.5	1.11	0.241	0.908	0.960	1.39	0.286	0.643	0.815	−3.98	0.362	0.403	0.679	−17.2	0.512	0.201	0.508
0.5	0.9	−2.99	0.321	0.935	1.017	−3.08	0.409	0.578	0.763	−21.6	0.560	0.308	0.570	−17.7	0.563	0.305	0.552
	0.5	−1.61	0.361	1.067	1.008	−6.31	0.377	0.982	1.004	−10.8	0.512	0.531	0.767	−27.0	0.543	0.472	0.700
*p*_*y*0_ = 0.3																	
0.1	0.9	1.67	0.190	1.009	0.949	0.34	0.223	0.734	0.855	7.26	0.297	0.413	0.683	−1.46	0.333	0.329	0.569
	0.5	4.83	0.219	1.004	1.049	−0.75	0.226	0.941	1.087	6.54	0.317	0.480	0.723	1.38	0.344	0.406	0.658
0.5	0.9	0.62	0.241	0.924	0.879	−0.27	0.274	0.717	0.814	−7.60	0.386	0.360	0.588	2.02	0.357	0.421	0.626
	0.5	1.73	0.248	0.918	0.957	−0.81	0.240	0.982	1.035	−1.15	0.369	0.416	0.664	−1.01	0.369	0.415	0.675
*p*_*y*0_ = 0.75																	
0.1	0.9	1.87	0.206	0.917	0.950	0.88	0.227	0.833	0.840	4.11	0.360	0.525	0.585	9.61	0.405	0.467	0.532
	0.5	2.44	0.273	0.978	0.960	3.79	0.303	0.881	0.887	15.8	0.473	0.564	0.573	11.2	0.439	0.608	0.628
0.5	0.9	−3.28	0.248	0.940	0.907	−0.05	0.267	0.873	0.872	0.09	0.390	0.597	0.601	24.4	0.451	0.517	0.530
	0.5	8.79	0.301	1.010	1.030	4.04	0.313	0.974	0.905	6.89	0.465	0.656	0.601	7.05	0.445	0.685	0.699

*Note*: Exposure misclassification rates were fixed at FPR_0_(X*) = 0.1 and TPR_0_(X*) = 0.9. The % Bias and SE are, respectively, the empirical percent bias and standard error of the MLE values. RE or RI below 1 indicates an efficiency loss compared to the optMLE design. The grid search successfully located the optMLE and optMLE-2 designs in all and ≥95% of replicates per setting, respectively; across all settings, 162 (1.4%) problematic replicates of the optMLE-2 design were discarded out of 12,000. Fewer than 1% and 5% of the replicates were discarded because of unstable estimates under the SRS, CC*, or BCC* designs when *p*_*y*0_ = 0.1 and 0.9, respectively. All other entries are based on 1000 replicates.

**Table 2:** Simulation results under outcome and exposure misclassification with varied exposure misclassification rates.

Exposure misclassification		optMLE-2				BCC*				CC*				SRS			
FPR*_0_*(X*)	TPR*_0_*(X*)	% Bias	SE	RE	RI	% Bias	SE	RE	RI	% Bias	SE	RE	RI	% Bias	SE	RE	RI
							*p_x_* = 0.1										
0.1	0.9	1.67	0.190	1.009	0.949	0.34	0.223	0.734	0.855	7.27	0.297	0.413	0.683	−1.46	0.333	0.329	0.569
	0.5	4.17	0.218	1.000	0.990	0.92	0.247	0.781	0.860	3.39	0.336	0.420	0.600	−1.54	0.338	0.414	0.637
0.5	0.9	1.74	0.296	0.973	1.015	−2.07	0.351	0.691	0.866	3.79	0.342	0.730	0.885	−0.06	0.351	0.693	0.866
	0.5	2.75	0.343	1.020	1.027	0.19	0.342	1.028	0.993	2.45	0.348	0.993	1.026	4.72	0.357	0.942	0.997
*p_x_* = 0.9																	
0.1	0.9	2.08	0.189	0.851	0.940	−1.14	0.201	0.750	0.879	3.88	0.310	0.316	0.584	0.08	0.339	0.265	0.520
	0.5	−5.23	0.290	0.960	0.910	4.23	0.343	0.685	0.811	2.37	0.345	0.678	0.771	0.89	0.381	0.555	0.750
0.5	0.9	−2.00	0.221	0.977	0.920	2.64	0.264	0.681	0.838	−2.07	0.337	0.418	0.600	5.52	0.366	0.355	0.576
	0.5	5.88	0.364	1.008	0.984	7.80	0.366	0.996	0.975	2.12	0.360	1.029	0.983	12.0	0.387	0.890	0.941

*Note*: Outcome misclassification rates were fixed at FPR_00_(Y*) = 0.1 and TPR_00_(Y*) = 0.9. The % Bias and SE are, respectively, the empirical percent bias and standard error of the MLE values. RE or RI below 1 indicates an efficiency loss compared to the optMLE design. The grid search successfully located the optMLE and optMLE-2 designs in all and ≥99% of replicates per setting, respectively; across all settings, 10 (<0.1%) problematic replicates of the optMLE-2 design were discarded out of 8000. All other entries are based on 1000 replicates.

**Table 3:** Simulation results with two- or three-wave approximate optimal designs under outcome and exposure misclassification.

Outcome misclassification		optMLE-2				optMLE-3			
FPR*_00_*(Y*)	TPR*_00_*(Y*)	% Bias	SE	RE	RI	%Bias	SE	RE	RI
0.1	0.9	1.33	0.190	1.009	0.949	3.33	0.191	1.000	0.959
	0.5	5.00	0.219	1.004	1.049	1.33	0.217	1.028	1.060
0.5	0.9	0.67	0.241	0.924	0.879	−2.00	0.235	0.975	0.879
	0.5	1.67	0.248	0.918	0.957	2.00	0.251	0.899	0.943

*Note*: Outcome misclassification rates were varied and exposure misclassification rates were fixed at FPR_0_(X*) = 0.1 and TPR_0_(X*) = 0.9. The % Bias and SE are, respectively, the empirical bias and standard error of the MLE values. RE or RI below 1 indicates an efficiency loss compared to the optMLE design. The grid search successfully located the optMLE, optMLE-2, and optMLE-3 designs in all, ≥99%, and ≥98% of replicates per setting, respectively; across all settings, 24 (0.6%) and 43 (1.1%) problematic replicates of the optMLE-2 and optMLE-3 designs, respectively, were discarded out of 4000. All other entries are based on 1000 replicates.

**Table 4:** Simulation results when the misspecification models used in the optimal design are misspecified.

		optMLE*				optMLE-2				optMLE-2*			
*δ_1_*	*δ_2_*	%Bias	SE	RE	RI	%Bias	SE	RE	RI	%Bias	SE	RE	RI
Misspecified misclassification mechanism for Y* and X*													
−1.0	−1.0	2.13	0.286	0.927	0.985	0.42	0.290	0.903	0.914	2.61	0.280	0.964	1.011
−0.5	−0.5	6.20	0.252	1.025	1.008	−1.24	0.262	0.949	0.999	−1.44	0.261	0.956	0.970
0.0	0.0	−2.35	0.260	1.000	1.000	0.17	0.270	0.932	1.065	0.17	0.270	0.932	1.065
0.5	0.5	1.76	0.251	1.053	0.974	2.87	0.256	1.014	0.971	1.36	0.260	0.986	0.978
1.0	1.0	2.70	0.255	0.944	0.918	4.12	0.263	0.883	0.963	5.06	0.256	0.936	0.938
Misspecified misclassification mechanism for Y* only													
0.0	−1.0	0.16	0.266	0.991	0.911	−5.54	0.287	0.855	0.897	0.29	0.272	0.948	0.935
0.0	−0.5	4.76	0.305	0.865	0.889	−3.22	0.296	0.918	0.962	−3.74	0.295	0.922	0.962
0.0	0.0	−2.35	0.260	1.000	1.000	0.17	0.270	0.932	1.065	0.17	0.270	0.932	1.065
0.0	0.5	7.51	0.279	1.267	1.159	5.16	0.305	1.058	1.051	3.64	0.301	1.087	1.038
0.0	1.0	4.76	0.257	1.018	0.988	0.35	0.266	0.952	0.966	−1.65	0.258	1.011	0.976
Misspecified misclassification mechanism for X* only													
−1.0	0.0	−2.22	0.270	1.030	1.031	−8.65	0.266	1.059	1.001	−8.47	0.273	1.005	0.984
−0.5	0.0	−1.32	0.252	0.994	0.947	−1.12	0.266	0.897	0.910	−3.60	0.262	0.924	0.999
0.0	0.0	−2.35	0.260	1.000	1.000	0.17	0.270	0.932	1.065	0.17	0.270	0.932	1.065
0.5	0.0	−0.25	0.256	0.984	0.982	1.11	0.255	0.990	1.022	3.64	0.266	0.908	0.908
1.0	0.0	−4.66	0.255	1.046	1.035	−0.30	0.256	1.037	1.027	−6.65	0.258	1.019	0.994

*Note*: Misclassification rates were fixed at FPR(·) = 0.1 and TPR(·) = 0.9. The error-prone exposure and outcome were generated from models including the interaction terms *δ*_1_ *XZ* and *δ*_2_*XZ*, respectively, but the optMLE* and optMLE-2* designs assumed only main effects for these models. The % Bias and SE are, respectively, the empirical bias and standard error of the MLE values. RE or RI below 1 indicates an efficiency loss compared to the optMLE design. The optMLE and optMLE* designs were located in all replicates and the optMLE-2 and optMLE-2* designs were located in ≥98% and ≥99% of replicates per setting, respectively; 65 (0.5%) and 17 (0.1%) problematic replicates out of 13,000 were discarded for the optMLE-2 and optMLE-2* designs, respectively. All other entries are based on 1000 replicates.

**Table 5:** Estimates and standard errors from the analysis of the VCCC dataset under the optMLE-2, BCC*, CC*, and SRS validation designs.

	Intercept		ART Status		$\sqrt{CD4}$	
Design	Log odds	SE	Log OR	SE	Log OR	SE
*Full cohort analyses*						
Gold standard	−1.184	0.294	0.032	0.260	−0.180	0.022
Naive	−0.043	0.234	−0.308	0.200	−0.148	0.015
*Two-phase analyses*						
Sampling strata defined by ADE and ART status						
SRS	−1.050	0.821	−0.161	0.996	−0.189	0.058
CC*	−1.527	0.438	0.090	0.394	−0.149	0.034
BCC*	−1.325	0.420	0.006	0.396	−0.169	0.035
optMLE-2	−1.542	0.394	0.118	0.368	−0.151	0.035
Sampling strata defined by ADE, ART status, and CD4 count						
BCC*	−1.402	0.421	0.115	0.406	−0.163	0.034
optMLE-2	−1.495	0.392	0.105	0.362	−0.150	0.034

*Note*: All results were averaged over 1000 replicates except for the SRS and optMLE-2 designs: SRS encountered 118 replicates where the MLE was unstable or did not converge because of the very small numbers of audited events or exposures, while the grid search algorithm failed to locate the optMLE-2 design in 40 and 48 of the replicates, respectively, under the first and second definitions of sampling strata.

## APPENDIX

### Derivations of ${\text{S}}^{\text{v}}(\cdot )$ and ${\text{S}}^{\bar{\text{v}}}(\cdot )$

Recall that Equation (1) is the joint distribution of a complete observation, which includes the validation indicator along with error-prone and error-free variables. Now, the joint distribution of just the error-prone and error-free variables, which is needed to derive the score vectors, is

$$\text{Pr}\left(Y^{*},X^{*},Y,X,Z\right)=\text{Pr}\left(Y^{*}|X^{*},Y,X,Z\right)\text{Pr}\left(X^{*}|Y,X,Z\right)\text{Pr}(Y|X,Z)\text{Pr}(X|Z)\text{Pr}(Z).$$

Then, depending on whether $V_{i}=1$ or 0,i∈{1,…,N}, we denote the log-likelihood contribution of the *i*th subject by $\ell^{\nu }\left(\theta ;Y_{i}^{*},X_{i}^{*},Y_{i},X_{i},Z_{i}\right)=ln\{{Pr}_{\theta }\left(Y_{i}^{*},X_{i}^{*},Y_{i},X_{i},Z_{i}\right)\}$ or $\ell^{\overset{‾}{v}}\left(\theta ;Y_{i}^{*},X_{i}^{*},Z_{i}\right)=ln\{{Pr}_{\theta }\left(Y_{i}^{*},X_{i}^{*},Z_{i}\right)\}=ln\{\sum_{y=0}^{1}\sum_{x=0}^{1}{Pr}_{\theta }\left(Y_{i}^{*},X_{i}^{*},y,x,Z_{i}\right)\}$, respectively. Recognize that $\ell^{v}\left(\theta ;Y_{i}^{*},X_{i}^{*},Y_{i},X_{i},Z_{i}\right)$ and $\ell^{\overset{‾}{v}}\left(\theta ;Y_{i}^{*},X_{i}^{*},Z_{i}\right)$ are equivalent to the summands in the first and second terms of the observed-data log likelihood from Equation (2).

Now, we denote the score vector for the *i*th subject based on their log-likelihood contribution by $S_{i}(\theta )={\left(S_{i}(\beta ),S_{i}(\eta {)}^{⊤}\right)}^{⊤}$, where

$$S_{i}\left(\theta_{j}\right)=V_{i}S^{\nu }\left(\theta_{j};Y_{i}^{*},X_{i}^{*},Y_{i},X_{i},Z_{i}\right)+\left(1-V_{i}\right)S^{\bar{v}}\left(\theta_{j};Y_{i}^{*},X_{i}^{*},Z_{i}\right)$$

and, for all $\theta_{j}\in \theta$,

$$S^{v}\left(\theta_{j};Y_{i}^{*},X_{i}^{*},Y_{i},X_{i},Z_{i}\right)=\frac{\partial }{\partial \theta_{j}}\ell^{v}\left(\theta ;Y_{i}^{*},X_{i}^{*},Y_{i},X_{i},Z_{i}\right)$$

and

$$S^{\bar{v}}\left(\theta_{j};Y_{i}^{*},X_{i}^{*},Z_{i}\right)=\frac{\partial }{\partial \theta_{j}}\ell^{\bar{v}}\left(\theta ;Y_{i}^{*},X_{i}^{*},Z_{i}\right).$$

We decompose $\eta^{⊤}$ into ${\left(\eta_{y^{*}}^{⊤},\eta_{x^{*}}^{⊤},\eta_{y}^{⊤},\eta_{x}^{⊤}\right)}^{⊤}$, where $\eta_{y^{*}},\eta_{x^{*}},\eta_{y}$, and $\eta_{x}$ correspond to the nuisance parameters in the ${Pr}_{\eta_{y^{*}}}\left(Y^{*}|X^{*},Y,X,Z\right),{Pr}_{\eta_{x^{*}}}\left(X^{*}|Y,X,Z\right),{Pr}_{\beta ,\eta_{y}}(Y|X,Z)$, and ${Pr}_{\eta_{x}}\left(X|Z\right)$ models, respectively. Then, we derive the elements of the score vectors as

$$S^{\nu }\left(\theta_{j};Y_{i}^{*},X_{i}^{*},Y_{i},X_{i},Z_{i}\right)=\{\begin{array}{ll} \left\{\frac{\partial }{\partial \theta_{j}}{\text{Pr}}_{\eta_{y^{*}}}\left(Y_{i}^{*}|X_{i}^{*},Y_{i},X_{i},Z_{i}\right)\right\}\;{\left\{{\text{Pr}}_{\eta_{y^{*}}}\left(Y_{i}^{*}|X_{i}^{*},Y_{i},X_{i},Z_{i}\right)\right\}}^{-1} & \;\text{if}\;\theta_{j}\in \eta_{y^{*}}\\ \left\{\frac{\partial }{\partial \theta_{j}}{\text{Pr}}_{\eta_{x^{*}}}\left(X_{i}^{*}|Y_{i},X_{i},Z_{i}\right)\right\}\;{\left\{{\text{Pr}}_{\eta_{x^{*}}}\left(X_{i}^{*}|Y_{i},X_{i},Z_{i}\right)\right\}}^{-1} & \;\text{if}\;\theta_{j}\in \eta_{x^{*}}\\ \left\{\frac{\partial }{\partial \theta_{j}}{\text{Pr}}_{\beta ,\eta_{y}}\left(Y_{i}|X_{i},Z_{i}\right)\right\}\;{\left\{{\text{Pr}}_{\beta ,\eta_{y}}\left(Y_{i}|X_{i}Z_{i}\right)\right\}}^{-1} & \;\text{if}\;\theta_{j}\in \left(\beta ,\eta_{y}\right)\\ \left\{\frac{\partial }{\partial \theta_{j}}{\text{Pr}}_{\eta_{x}}\left(X_{i}|Z_{i}\right)\right\}\;{\left\{{\text{Pr}}_{\eta_{x}}\left(X_{i}|Z_{i}\right)\right\}}^{-1} & \;\text{if}\;\theta_{j}\in \eta_{x} \end{array}$$

and

$$S^{\bar{v}}\left(\theta_{j};Y_{i}^{*},X_{i}^{*},Z_{i}\right)=\frac{\sum_{y=0}^{1}\sum_{x=0}^{1}\frac{\partial }{\partial \theta_{j}}\left\{{\text{Pr}}_{\eta_{y^{*}}}\left(Y_{i}^{*}|X_{i}^{*},y,x,Z_{i}\right){\text{Pr}}_{\eta_{x^{*}}}\left(X_{i}^{*}|y,x,Z_{i}\right){\text{Pr}}_{\beta ,\eta_{y}}\left(y|x,Z_{i}\right){\text{Pr}}_{\eta_{x}}\left(x|Z_{i}\right)\right\}}{\sum_{y=0}^{1}\sum_{x=0}^{1}{\text{Pr}}_{\eta_{y^{*}}}\left(Y_{i}^{*}|X_{i}^{*},y,x,Z_{i}\right){\text{Pr}}_{\eta_{x^{*}}}\left(X_{i}^{*}|y,x,Z_{i}\right){\text{Pr}}_{\beta ,\eta_{y}}\left(y|x,Z_{i}\right){\text{Pr}}_{\eta_{x}}\left(x|Z_{i}\right)}.$$
