# Supplementary material for: Optimal multiwave validation of secondary use data with outcome and exposure misclassification
Source: Can J Stat. Author manuscript; Available in PMC 2025 Jun 1. (PMC11610482; doi:10.1002/cjs.11772)
Supplement: Supplemental Material [file NIHMS2035539-supplement-Supplemental_Material.pdf]

Supplementary Materials for "Optimal multiwave validation of secondary use data with outcome and exposure misclassification"

Sarah C. Lotspeich, Gustavo G. C. Amorim, Pamela A. Shaw, Ran Tao, and Bryan E. Shepherd

Appendix S1 Choosing Step Sizes for the Adaptive Grid Search

Our adaptive grid search algorithm locates the optimal design by searching a series of grids that are "adaptively" constructed at iteratively finer scales and over more focused candidate design spaces. The choice of the step sizes (or scales) of the grids appears inconsequential, but we detail the implementation used by our `auditDesignR` R package to suggest them. Our software assumes a user-specified, maximum-allowable grid size, which would be dictated by their machine; we use 10,000 as the maximum in Sections 3–5.

We want to choose the first step size $s^{(1)}$ to be the largest value for which the dimension of $\boldsymbol{G}^{(1)}$ still falls below the allowed maximum. Calculating the dimension of a grid based on a potential step size s involves applying the "stars and bars" problem from combinatorics. Based on the audit size constraint (Eq. (5) in the main text), the number of "stars" to partition is equal to $(n - Km)/s$, i.e., the number of subjects to allocate (in increments of the step size s) after the minimum m has been dispensed to each stratum, and there are $K - 1$ "bars" (i.e., partitions to form) between them. Thus, the dimension of the first grid (in number of rows) based on a step size of s is

$$\text{rows}\left\{\boldsymbol{G}^{(1)} \mid s\right\} = \binom{(n-Km)/s + (K-1)}{(K-1)}. \tag{S.1}$$

For simplicity, we start by considering all possible values s that share common factors. (This also ensures overlap between the neighbourhoods in successive iterations such that, as a safety net, no candidate designs are "left out.") In the example from Section 2.3 with $n = 400$, $K = 4$, and $m = 10$, we consider possible step sizes $s \in \{180, 90, 45, 15, 5, 1\}$, which leads to possible grids with rows$\left\{\boldsymbol{G}^{(1)} \mid \boldsymbol{s}\right\} = \{10, 35, 165, 2925, 67525, 7906261\}$, respectively, following Equation (S.1). Thus, we select $s^{(1)} = 15$, since it is the largest

potential step size to keep the grid smaller than 10,000 rows.

There is slightly more to consider in choosing step sizes $s^{(t)}$ for successive iterations (i.e., $t > 1$). We still want to cover the entire candidate design space in an efficient way. On top of that, the candidate design space has now narrowed to be in the $s^{(t-1)}$-person window around the last iteration's "best" design, so based on $\{n_{y^*x^*\boldsymbol{z}}^{(t-1)}\}$ we want to impose lower and upper bounds on the stratum sizes considered. Calculating the size of a grid based on a possible step size of s where $n_{y^*x^*z}^{(t)} \geq (n_{y^*x^*\boldsymbol{z}}^{(t-1)} - s^{(t-1)})$ (i.e., all candidate designs are above the lower-bound of the neighbourhood) involves a modification to Equation (S.1):

$$\text{rows}\left\{\boldsymbol{G}^{(t)} \mid s, s^{(t-1)}, n_{y^*x^*\boldsymbol{z}}^{(t)} \geq n_{y^*x^*\boldsymbol{z}}^{(t-1)}\right\} = \binom{(n-Km)/s + (K-1) - \sum_{y^*=0}^{1}\sum_{x^*=0}^{1}\sum_{\boldsymbol{z}}(n_{y^*x^*\boldsymbol{z}}^{(t-1)} - s^{(t-1)})/s}{(K-1)}. \tag{S.2}$$

Still, we need to subtract from Equation (S.2) the number of candidate designs where the stratum sizes are above the upper bound of the neighbourhood. In `auditDesignR`, we manually tabulate the number of such designs and subtract it from Equation (S.2) to calculate the expected grid size $\text{rows}\left\{\boldsymbol{G}^{(t)} \mid s, s^{(t-1)}, n_{y^*x^*\boldsymbol{z}}^{(t-1)}\right\}$ for step s in iteration t. As before, we consider possible values in $\boldsymbol{s}$ that share common factors, but now we also want to focus on $\boldsymbol{s} < s^{(t-1)}$. In the second iteration of the example from Section 2.3, we consider $\boldsymbol{s} = \{5, 1\}$, which are expected to lead to grids with $\text{rows}\left\{\boldsymbol{G}^{(t)} \mid \boldsymbol{s}, s^{(t-1)}, n_{y^*x^*\boldsymbol{z}}^{(t-1)}\right\} = \{134, 10296\}$, respectively. Thus, we select $s^{(2)} = 5$ since it is the largest (and only) step size considered that keeps the grid smaller than 10,000 rows. This process is repeated until we can reasonably reach a step size of $s^{(T)} = 1$ while keeping the size of the grid below the maximum.

Appendix S2 Additional Simulations, Tables, and Figures

Table S1. Three versions of the optimal design under outcome and exposure misclassification.

(a) Exposure misclassification rates fixed at $\mathrm{FPR}_0(X^*) = 0.1$ and $\mathrm{FPR}_0(X^*) = 0.9$											
**Outcome Misclassification**		**optMLE**		**optMLE-EXP**				**optMLE-FC**			
$\mathbf{FPR_{00}}(Y^*)$	$\mathbf{TPR_{00}}(Y^*)$	% Bias	SE	% Bias	SE	RE	RI	% Bias	SE	RE	RI
0.1	0.9	−1.999	0.191	−2.033	0.193	0.977	0.983	0.198	0.192	0.987	0.976
	0.5	1.020	0.220	4.607	0.225	0.956	1.019	2.340	0.215	1.039	1.012
0.5	0.9	1.500	0.232	2.659	0.221	1.094	0.940	2.183	0.222	1.093	1.024
	0.5	4.594	0.238	3.191	0.239	0.988	0.949	6.363	0.248	0.921	0.982
**(b) Outcome misclassification rates fixed at $\mathrm{FPR}_{00}(Y^*) = 0.1$ and $\mathrm{FPR}_{00}(Y^*) = 0.9$**											
**Exposure Misclassification**		**optMLE**		**optMLE-EXP**				**optMLE-FC**			
$\mathbf{FPR_0}(X^*)$	$\mathbf{TPR_0}(X^*)$	% Bias	SE	% Bias	SE	RE	RI	% Bias	SE	RE	RI
0.1	0.9	−1.999	0.191	−2.033	0.193	0.977	0.983	0.198	0.192	0.987	0.976
	0.5	5.078	0.218	4.227	0.213	1.048	1.002	8.684	0.216	1.013	0.967
0.5	0.9	4.508	0.292	8.276	0.287	1.033	1.032	4.243	0.285	1.051	0.995
	0.5	2.824	0.347	8.117	0.341	1.031	1.007	0.796	0.338	1.054	1.057

The optMLE design was based on the true parameters $\boldsymbol{\theta}$ and the observed stratum sizes $\{N_{y^*x^*}\}$ in each replicate. The optMLE-EXP design was based on the true parameters $\boldsymbol{\theta}$ and the expected stratum sizes $\mathrm{E}(N_{y^*x^*}) = N \times \Pr(Y^* = y^*, X^* = x^*)$; this design was the same for each replicate. The optMLE-FC design was based on the full-cohort parameter estimates $\hat{\boldsymbol{\theta}}$ and the observed stratum sizes $\{N_{y^*x^*}\}$ in each replicate. The % Bias and SE are, respectively, the empirical percent bias and standard error of the MLE values. Each entry is based on 1,000 replicates.

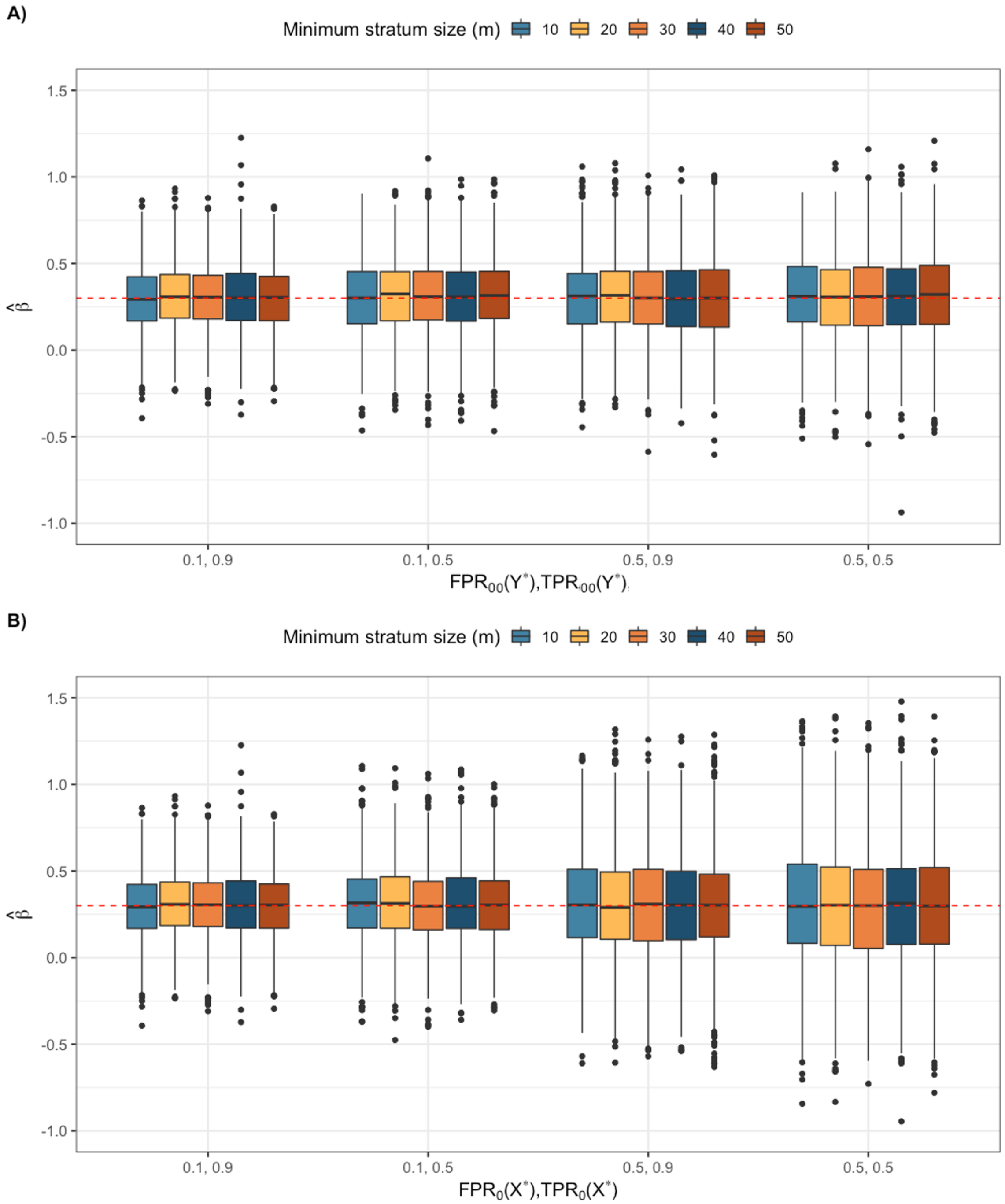

Figure S1. Distribution of $\hat{\beta}$ under the optMLE design with outcome and exposure misclassification. The dashed lines denote the true value $\beta = 0.3$. Exposure and outcome misclassification rates are fixed at $\text{FPR}_0(X^*) = 0.1$ and $\text{TPR}_0(X^*) = 0.9$ in **(A)** and $\text{FPR}_{00}(Y^*) = 0.1$ and $\text{TPR}_{00}(Y^*) = 0.9$ in **(B)**.

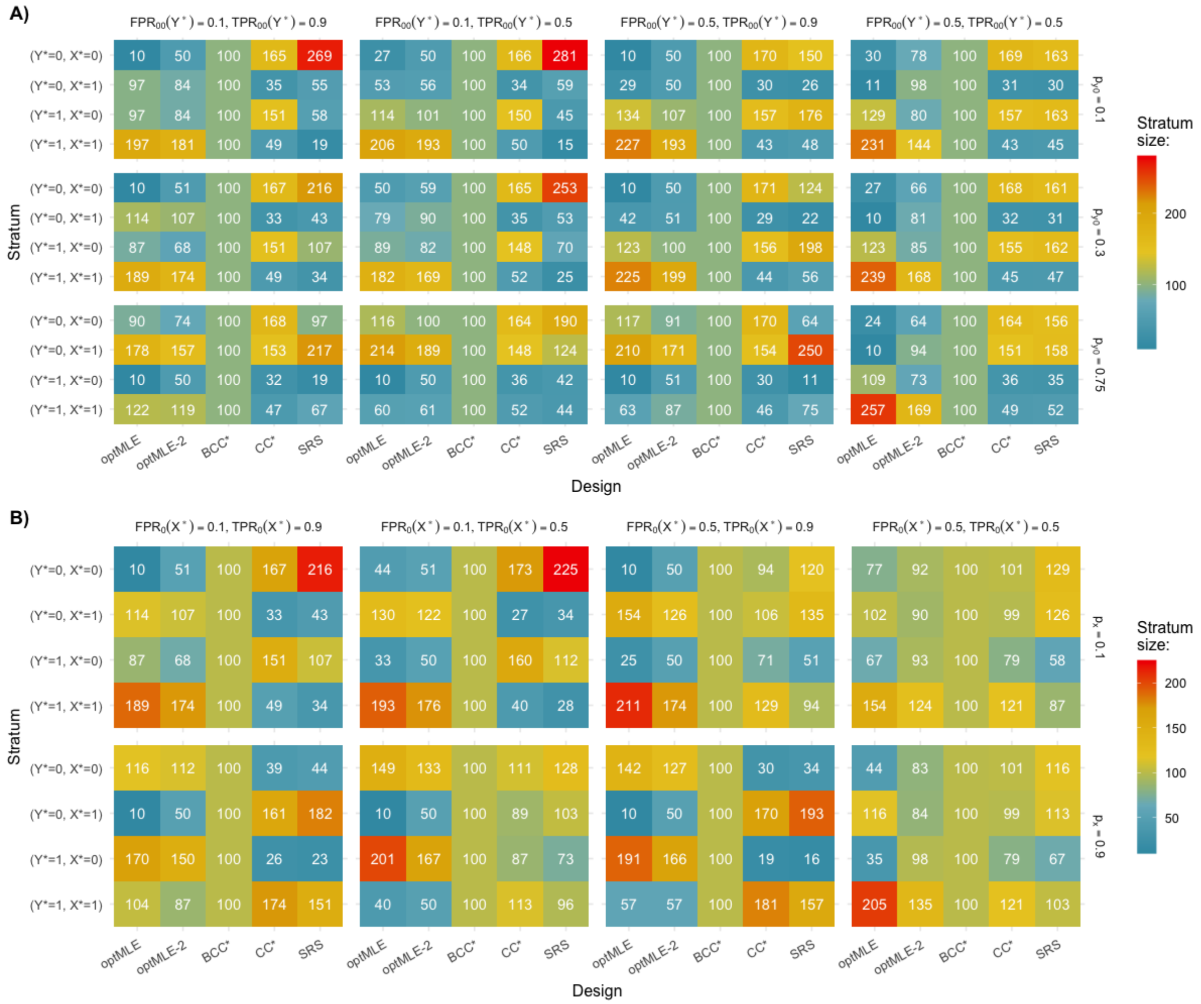

Figure S2. Average Phase II stratum sizes $\{n_{y^*x^*}\}$ under outcome and exposure misclassification. Exposure and outcome misclassification rates are fixed at $\text{FPR}_0(X^*) = 0.1$ and $\text{TPR}_0(X^*) = 0.9$ in **(A)** and $\text{FPR}_{00}(Y^*) = 0.1$ and $\text{TPR}_{00}(Y^*) = 0.9$ in **(B)**.

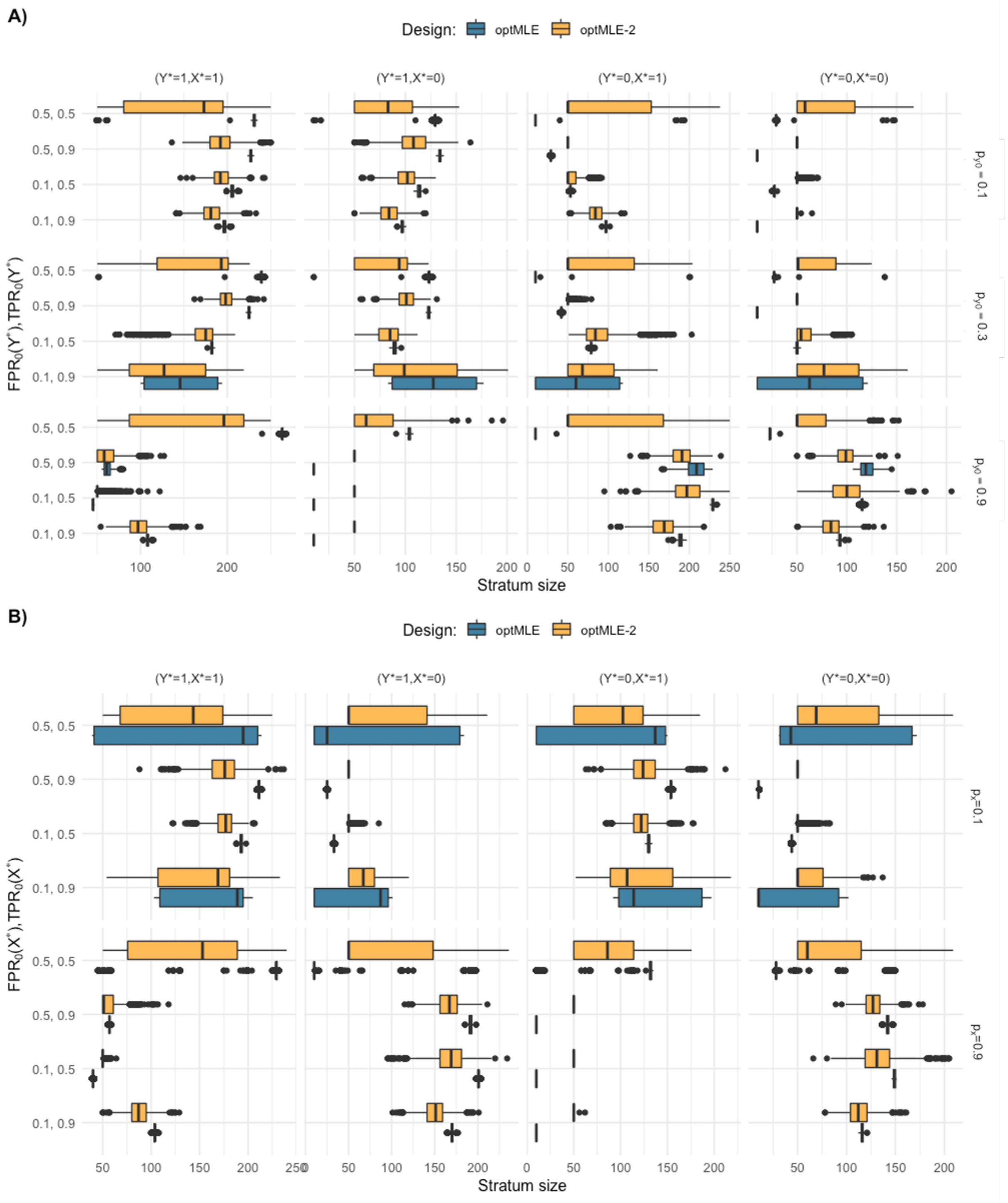

Figure S3. Distribution of Phase II stratum sizes $\{n_{y^*x^*}\}$ under outcome and exposure misclassification. Exposure and outcome misclassification rates are fixed at $\mathrm{FPR}_0(X^*) = 0.1$ and $\mathrm{TPR}_0(X^*) = 0.9$ in **(A)** and $\mathrm{FPR}_{00}(Y^*) = 0.1$ and $\mathrm{TPR}_{00}(Y^*) = 0.9$ in **(B)**.

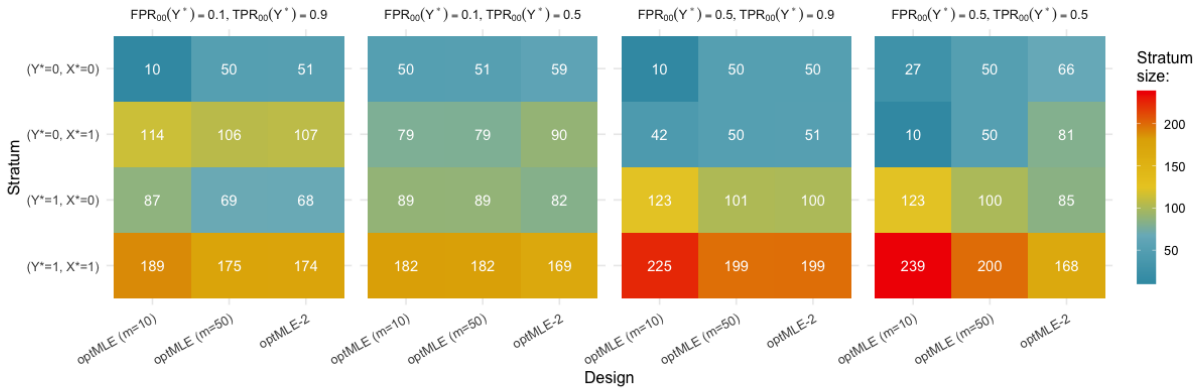

Figure S4. Distribution of Phase II stratum sizes $\{n_{y^*x^*}\}$ under outcome and exposure misclassification. Two versions of the optMLE design were considered (requiring minimum stratum sizes of $m = 10$ or 50), alongside the approximate, two-wave optMLE-2 design. Exposure misclassification rates were fixed at $\text{FPR}_0(X^*) = 0.1$ and $\text{TPR}_0(X^*) = 0.9$.

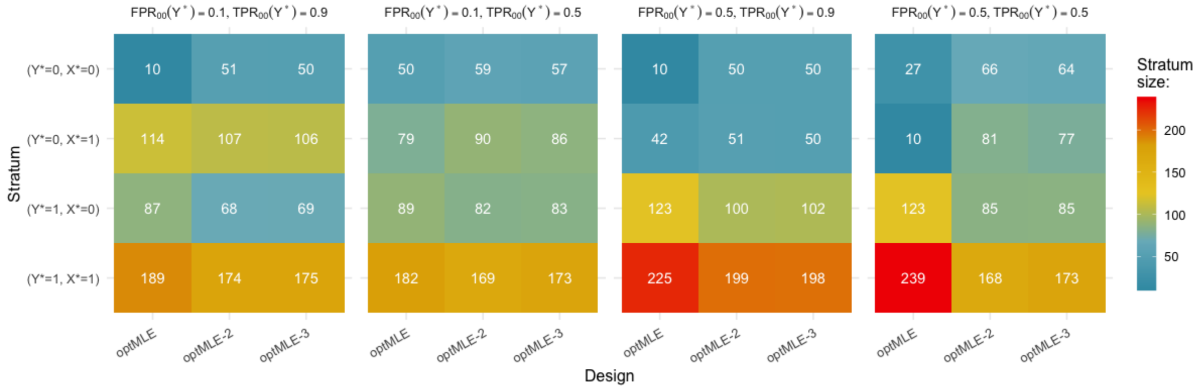

Figure S5. Distribution of Phase II stratum sizes $\{n_{y^*x^*}\}$ under outcome and exposure misclassification. The optMLE design was compared to approximate, two-wave and three-wave optMLE-2 and optMLE-3 designs, respectively. Exposure misclassification rates were fixed at $\text{FPR}_0(X^*) = 0.1$ and $\text{TPR}_0(X^*) = 0.9$.

Appendix S2.1 Incorporating an Additional Error-Free Covariate

We simulated data using Equation (1) for a Phase I sample of $N = 10{,}000$ subjects. We generated an error-free binary covariate Z from a Bernoulli distribution with $\Pr(Z = 1) \equiv p_z = 0.25$ or 0.5. We generated X and Y from Bernoulli distributions with $\Pr(X = 1 \mid Z) = [1 + \exp\{-(-2.2 + 0.5Z)\}]^{-1}$ and $\Pr(Y = 1 \mid X, Z) = [1 + \exp\{-(-0.85 + 0.3X + \beta_z Z)\}]^{-1}$, where $\beta_z = -0.25$, 0, or 0.25. We set the misclassification rates at $\text{FPR}_0(X^*) = \text{FPR}_{00}(Y^*) = 0.25$ and $\text{TPR}_0(X^*) = \text{TPR}_{00}(Y^*) = 0.75$, such that X^* and Y^* were generated from Bernoulli distributions with $\Pr(X^* = 1 \mid Y, X, Z) = [1 + \exp\{-(-1.1 + 0.45Y + 2.2X + \lambda Z)\}]^{-1}$ and $\Pr(Y^* = 1 \mid X^*, Y, X, Z) = [1 + \exp\{-(-1.1 + 0.275X^* + 2.2Y + 0.275X + \lambda Z)\}]^{-1}$, with $\lambda = -1$, 0, or 1. We defined eight sampling strata based on (Y^*, X^*, Z) and selected $n = 400$ subjects in Phase II. As in Section 3.2, we set $m = 10$, but it is worth noting that larger choices of m can quickly eat into our efficiency gain since we have twice as many strata. (In fact, $m = 50$ forces the optimal designs to be equal to the BCC* design.) The grid search parameters varied between replicates, but the most common choices were a six-iteration grid search with step sizes $\boldsymbol{s} = \{40, 20, 10, 5, 2, 1\}$ and a five-iteration grid search with step sizes $\boldsymbol{s} = \{25, 8, 5, 2, 1\}$ to locate the optMLE and optMLE-2 designs, respectively.

Table S2 shows simulation results for the MLE under these designs. The optMLE-2 design continued to be highly efficient, with gains as high as 43%, 56%, and 59% over the BCC*, CC*, and SRS designs, respectively. Figure S6 shows the average Phase II stratum sizes of the designs under these settings. The results were similar with either a 25% or 50% prevalence of $Z = 1$. The optimal designs favoured subjects with $Z = 1$, which was partly because $\text{Var}(X \mid Z = 1)$ was larger than $\text{Var}(X \mid Z = 0)$, such that the true value of X was harder to "guess" when $Z = 1$ and validating X among subjects with

$Z = 1$ was more “rewarding” than validating X among subjects with $Z = 0$.

Table S2. Simulation results under outcome and exposure misclassification with an additional error-free covariate.

		**optMLE-2**				**BCC***				**CC***				**SRS**			
λ	β_Z	**% Bias**	**SE**	**RE**	**RI**	**% Bias**	**SE**	**RE**	**RI**	**% Bias**	**SE**	**RE**	**RI**	**% Bias**	**SE**	**RE**	**RI**
								$\Pr(Z=1)=0.25$									
−1	−0.25	−4.095	0.225	1.329	1.246	−1.728	0.296	0.767	0.835	−5.322	0.321	0.653	0.833	−12.342	0.352	0.543	0.770
	0.00	0.009	0.227	1.097	1.024	−0.734	0.288	0.683	0.792	3.217	0.325	0.535	0.734	−3.221	0.332	0.515	0.755
	0.25	0.993	0.221	1.036	0.991	7.537	0.287	0.613	0.764	−1.844	0.333	0.454	0.681	0.261	0.337	0.444	0.682
0	−0.25	−2.982	0.249	0.975	0.960	−1.728	0.296	0.690	0.790	−5.322	0.321	0.587	0.788	−12.342	0.352	0.489	0.729
	0.00	−1.351	0.245	1.021	0.965	−0.734	0.288	0.735	0.816	3.218	0.325	0.577	0.756	−3.223	0.332	0.554	0.778
	0.25	0.023	0.242	0.879	0.925	7.537	0.287	0.625	0.787	−1.844	0.333	0.463	0.701	0.261	0.337	0.453	0.702
1	−0.25	−4.095	0.267	0.943	1.000	−1.728	0.296	0.767	0.835	−5.322	0.321	0.653	0.833	−12.341	0.352	0.543	0.770
	0.00	−5.531	0.275	0.840	0.940	−0.734	0.288	0.766	0.857	3.216	0.325	0.601	0.794	−3.221	0.332	0.578	0.816
	0.25	−2.354	0.270	0.932	1.065	7.538	0.287	0.823	0.966	−1.844	0.333	0.610	0.860	0.261	0.337	0.596	0.861
								$\Pr(Z=1)=0.50$									
−1	−0.25	−4.512	0.229	1.295	1.241	0.669	0.304	0.735	0.971	−2.470	0.310	0.707	0.913	−3.921	0.338	0.596	0.832
	0.00	−3.361	0.228	1.088	1.093	−1.451	0.264	0.811	0.902	−0.474	0.312	0.579	0.764	−6.093	0.317	0.559	0.764
	0.25	−1.931	0.225	0.994	0.972	4.339	0.287	0.612	0.776	−1.055	0.308	0.531	0.685	1.577	0.304	0.545	0.715
0	−0.25	0.035	0.250	0.927	1.052	0.669	0.304	0.628	0.869	−2.470	0.310	0.603	0.817	−3.921	0.338	0.509	0.745
	0.00	4.706	0.239	0.911	0.947	−1.451	0.264	0.752	0.838	−0.473	0.312	0.538	0.710	−6.092	0.317	0.519	0.710
	0.25	3.039	0.251	0.833	1.008	4.339	0.287	0.636	0.838	−1.055	0.308	0.553	0.739	1.577	0.304	0.567	0.771
1	−0.25	−4.512	0.277	0.890	1.029	0.669	0.304	0.735	0.971	−2.470	0.310	0.707	0.913	−3.921	0.338	0.596	0.832
	0.00	0.756	0.267	0.991	0.994	−1.452	0.264	1.014	1.012	−0.474	0.312	0.725	0.858	−6.093	0.317	0.700	0.857
	0.25	2.724	0.261	0.916	0.939	4.339	0.287	0.760	0.867	−1.054	0.308	0.660	0.765	1.577	0.304	0.678	0.799

The % Bias and SE are, respectively, the empirical percent bias and standard error of the MLE. The grid search algorithm successfully located the optMLE and optMLE-2 designs in all and >99% of replicates per setting, respectively; 16 (<0.1%) problematic replicates out of 18,000 were discarded. All other entries are based on 1,000 replicates.

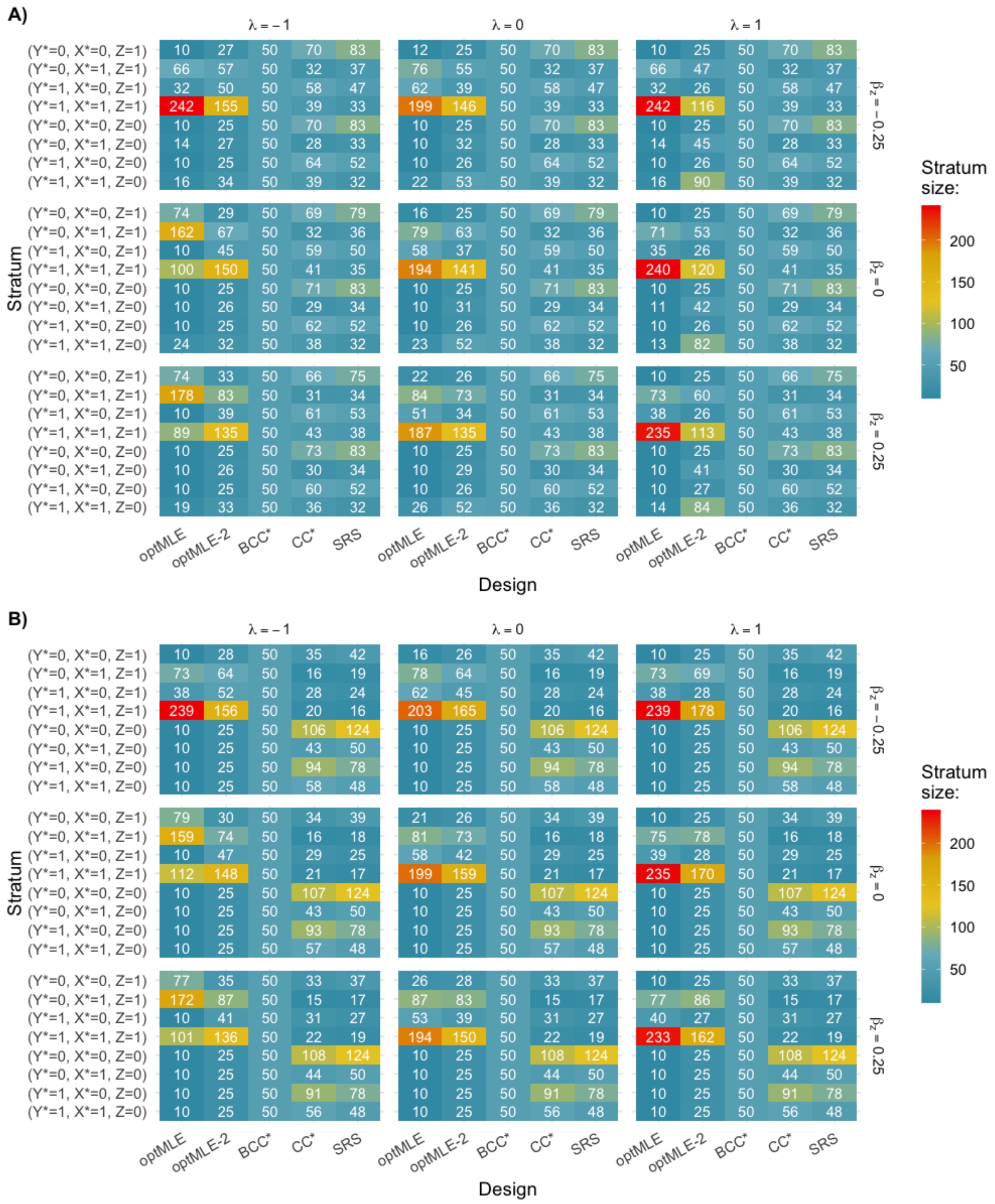

Figure S6. Average Phase II stratum sizes $n_{y^*x^*z}$ under outcome and exposure misclassification when an error-free binary covariate Z with **(A)** 25% or **(B)** 50% prevalence was used in sampling.

Appendix S2.2 Outcome Misclassification Only

For the special scenario of outcome misclassification alone, $X^* = X$ such that Equation (1) reduces to $\Pr(Y^*, Y, X) = \Pr(Y^* \mid Y, X) \Pr(Y \mid X) \Pr(X)$. We generated Y and X in the same way as in Section 3.2, with $p_{y0} = 0.3$ and $p_x = 0.1$, for a sample of $N = 10{,}000$ subjects. We generated error-prone Y^* from a Bernoulli distribution with $\Pr(Y^* = 1 \mid Y, X) = [1 + \exp\{-(\alpha_0 + \alpha_1 Y + 0.28X)\}]^{-1}$, where α_0 and α_1 were defined in the same way as in Section 3.2 with $\text{FPR}_0(Y^*) \in \{0.1, 0.5\}$ and $\text{TPR}_0(Y^*) \in \{0.9, 0.5\}$. Note that without X^*, the baseline false positive and true positive rates for Y^* are defined with $X = 0$ in this setting (hence the single zero subscript). We set $n = 400$. Without exposure misclassification, the sampling strata for the BCC*, optMLE, and optMLE-2 designs were defined by (Y^*, X). Each setting was replicated 1,000 times.

Simulation results for the MLE are included in Table S4(a). The optMLE-2 design did not lose much efficiency relative to the optMLE design and typically surpassed the efficiencies of the BCC*, CC*, and SRS designs, with gains as high as 21%, 72%, and 74%, respectively. Figure S8 shows the average Phase II stratum sizes selected under each of the designs. The optimal designs favoured strata with the less-frequent value of Y^* in all settings where Y^* was informative, i.e., with $\text{FPR}_0(Y^*) \neq 0.5$ or $\text{TPR}_0(Y^*) \neq 0.5$. In the highest-error setting, the optimal designs appeared to be similar to the BCC* design.

Appendix S2.3 Exposure Misclassification Only

For the special scenario of exposure misclassification alone, $Y^* = Y$ such that Equation (1) reduces to $\Pr(X^*, Y, X) = \Pr(X^* \mid Y, X) \Pr(Y \mid X) \Pr(X)$. We generated Y and X in the same way as in Appendix S2.2 for a Phase I sample of $N = 10{,}000$ subjects. We generated error-prone X^* from a Bernoulli distribution with $\Pr(X^* = 1 \mid Y, X) = [1 + \exp\{-(\gamma_0 + 0.45Y + \gamma_1 X)\}]^{-1}$, where γ_0 and γ_1 were defined in the same way as in Section 3.2 with

$\mathrm{FPR}_0(X^*) \in \{0.1, 0.5\}$ and $\mathrm{TPR}_0(X^*) \in \{0.9, 0.5\}$. We set $n = 400$. Without outcome misclassification, the sampling strata for the BCC*, optMLE, and optMLE-2 designs were defined by (Y, X^*). Results in each setting are based on 1,000 replicates.

Simulation results for the MLE are included in Table S4(b). The optMLE-2 design did not lose much efficiency relative to the optMLE design and typically surpassed the efficiencies of the BCC*, CC*, and SRS designs, with gains as high as 32%, 69% and 69%, respectively. Figure S9 shows the average Phase II stratum sizes selected under each of the designs. The optimal designs favoured strata with the less-frequent value of X^* in all settings where X^* was informative, i.e., with $\mathrm{FPR}_0(X^*) \neq 0.5$ or $\mathrm{TPR}_0(X^*) \neq 0.5$. In the highest-error setting, the optimal designs appeared to be similar to the BCC* design. Together with Appendix S2.2, these results suggest that the optimal designs seemed to target the less-frequent value of the error-prone variable with very little regard for the error-free variable.

Table S3. Additional simulation results for other designs when the misspecification models used in the optimal design are misspecified.

		BCC*				CC*				SRS			
δ_1	δ_2	% Bias	SE	RE	RI	% Bias	SE	RE	RI	% Bias	SE	RE	RI
**Misspecified misclassification mechanism for Y^* and X^***													
−1.0	−1.0	−0.570	0.317	0.754	0.893	2.058	0.336	0.672	0.804	1.369	0.364	0.572	0.783
−0.5	−0.5	−0.053	0.310	0.674	0.903	2.121	0.326	0.610	0.819	−7.261	0.348	0.537	0.759
0.0	0.0	7.538	0.287	0.823	0.966	−1.844	0.333	0.610	0.860	0.261	0.337	0.596	0.861
0.5	0.5	5.243	0.318	0.657	0.790	−1.785	0.318	0.656	0.805	−2.903	0.358	0.519	0.710
1.0	1.0	−10.917	0.320	0.599	0.779	−1.241	0.339	0.533	0.712	−7.684	0.339	0.533	0.710
**Misspecified misclassification mechanism for Y^***													
0.0	−1.0	3.504	0.314	0.712	0.795	−3.587	0.336	0.622	0.749	−6.257	0.344	0.595	0.812
0.0	−0.5	−1.159	0.315	0.806	0.912	2.174	0.346	0.671	0.775	1.268	0.363	0.610	0.782
0.0	0.0	7.538	0.287	0.823	0.966	−1.844	0.333	0.610	0.860	0.261	0.337	0.596	0.861
0.0	0.5	0.570	0.312	1.011	1.001	−3.129	0.323	0.946	0.993	−2.122	0.347	0.817	0.890
0.0	1.0	−6.256	0.306	0.716	0.884	2.449	0.336	0.597	0.733	−8.055	0.354	0.536	0.719
**Misspecified misclassification mechanism for X^***													
−1.0	0.0	−11.340	0.299	0.837	0.968	−7.776	0.333	0.674	0.804	−2.565	0.333	0.675	0.846
−0.5	0.0	−2.549	0.318	0.625	0.807	−7.388	0.330	0.583	0.750	−9.968	0.343	0.538	0.752
0.0	0.0	7.538	0.287	0.823	0.966	−1.844	0.333	0.610	0.860	0.261	0.337	0.596	0.861
0.5	0.0	−2.139	0.316	0.646	0.836	0.820	0.340	0.559	0.783	−3.184	0.344	0.545	0.786
1.0	0.0	−2.764	0.326	0.638	0.784	6.405	0.322	0.653	0.759	−1.454	0.342	0.581	0.803

The error-prone exposure and outcome were generated following $\Pr(X^* = 1 \mid Y, X, Z) = [1 + \exp\{-(-1.1 + 0.45Y + 2.2X + Z + \delta_1 XZ)\}]^{-1}$ and $\Pr(Y^* = 1 \mid X^*, Y, X, Z) = [1 + \exp\{-(-1.1 + 0.275X^* + 2.2Y + 0.275X + Z + \delta_2 XZ)\}]^{-1}$. The % Bias and SE are, respectively, the empirical percent bias and standard error of the MLE values. All entries are based on 1,000 replicates.

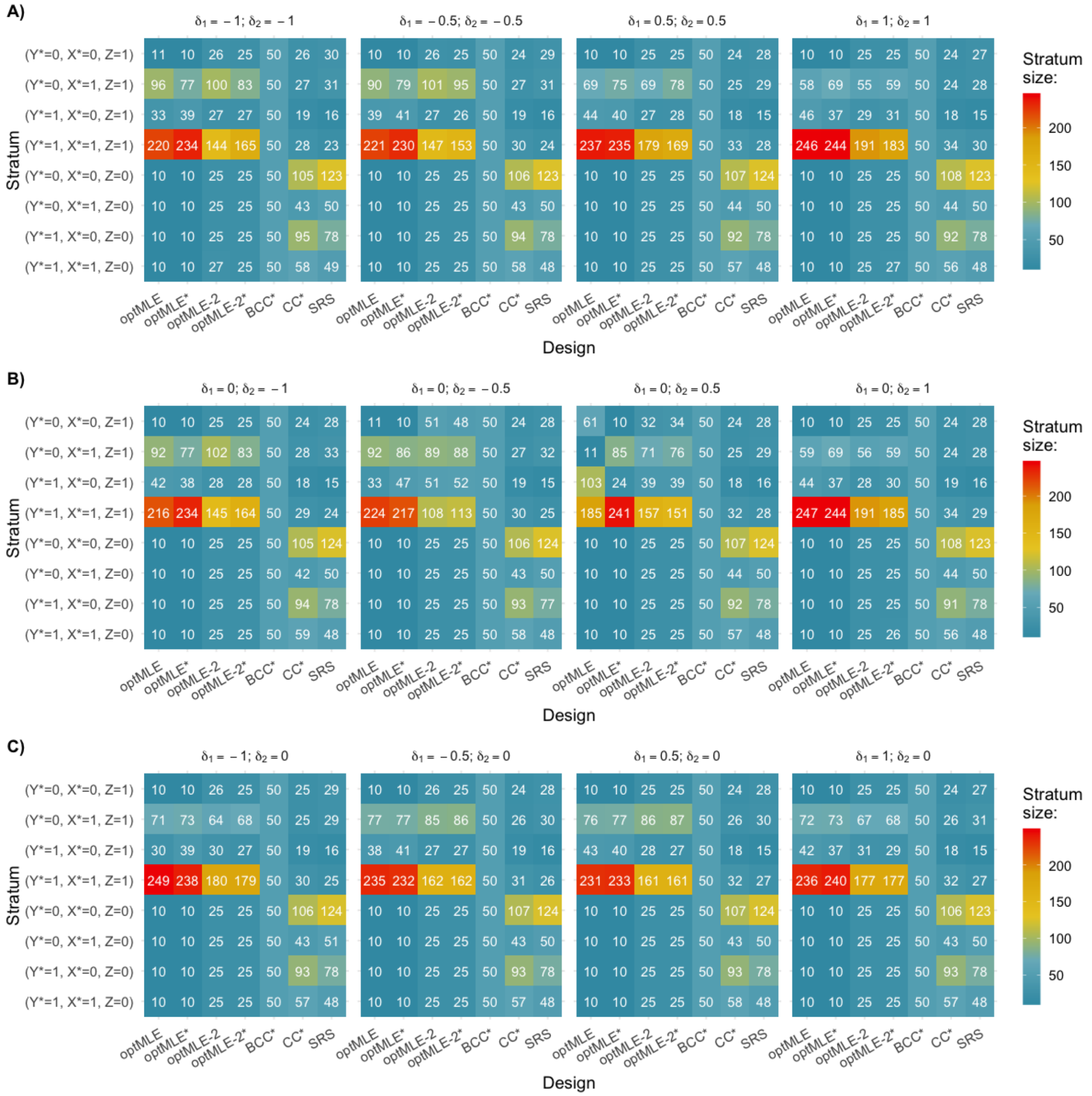

Figure S7. Average Phase II stratum sizes $\{n_{y^*x^*z}\}$ when optimal designs optMLE* and optMLE-2* can be derived based on misspecified misclassification mechanisms. In **(A)**, **(B)**, and **(C)**, the Y^* and X^*, Y^* only, and X^* only misclassification mechanisms are misspecified, respectively.

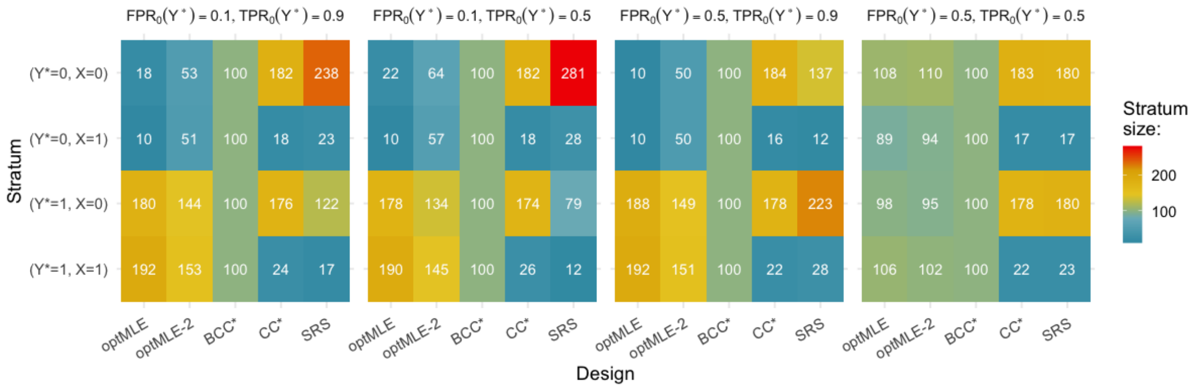

Figure S8. Average Phase II stratum sizes $\{n_{y^*x}\}$ under outcome misclassification.

Table S4. Simulation results under either outcome or exposure misclassification.

(a) Outcome Misclassification Only

Misclassification		optMLE-2				BCC*				CC*				SRS			
**FPR$_0$(Y^*)**	**TPR$_0$(Y^*)**	% Bias	SE	RE	RI	% Bias	SE	RE	RI	% Bias	SE	RE	RI	% Bias	SE	RE	RI
0.1	0.9	2.066	0.119	0.935	0.965	−0.752	0.133	0.741	0.800	1.635	0.209	0.302	0.526	0.881	0.234	0.240	0.452
	0.5	1.158	0.179	0.866	0.991	−0.696	0.192	0.754	0.902	−2.929	0.293	0.325	0.563	3.086	0.315	0.281	0.548
0.5	0.9	−0.081	0.199	0.859	0.898	−0.494	0.218	0.717	0.790	−4.366	0.376	0.241	0.477	2.088	0.345	0.287	0.519
	0.5	3.171	0.219	0.914	0.997	1.461	0.207	1.019	1.061	−2.941	0.373	0.314	0.623	0.319	0.348	0.362	0.657

(b) Exposure Misclassification Only

Misclassification		optMLE-2				BCC*				CC*				SRS			
**FPR$_0$(X^*)**	**TPR$_0$(X^*)**	% Bias	SE	RE	RI	% Bias	SE	RE	RI	% Bias	SE	RE	RI	% Bias	SE	RE	RI
0.1	0.9	−1.716	0.158	0.861	0.917	3.146	0.180	0.660	0.838	1.821	0.282	0.270	0.536	1.134	0.285	0.264	0.540
	0.5	0.892	0.206	1.002	0.928	4.337	0.237	0.754	0.788	2.750	0.313	0.433	0.607	0.785	0.332	0.385	0.618
0.5	0.9	−3.594	0.298	0.852	0.902	2.773	0.361	0.580	0.736	−3.331	0.327	0.706	0.831	−4.604	0.336	0.667	0.795
	0.5	−0.987	0.345	0.941	0.957	5.740	0.343	0.949	0.959	2.027	0.348	0.923	0.964	−3.840	0.362	0.854	0.880

The % Bias and SE are, respectively, the empirical percent bias and standard error of the MLE values. The grid search algorithm successfully located the optMLE and optMLE-2 designs in all settings and replicates. Each entry is based on 1,000 replicates.

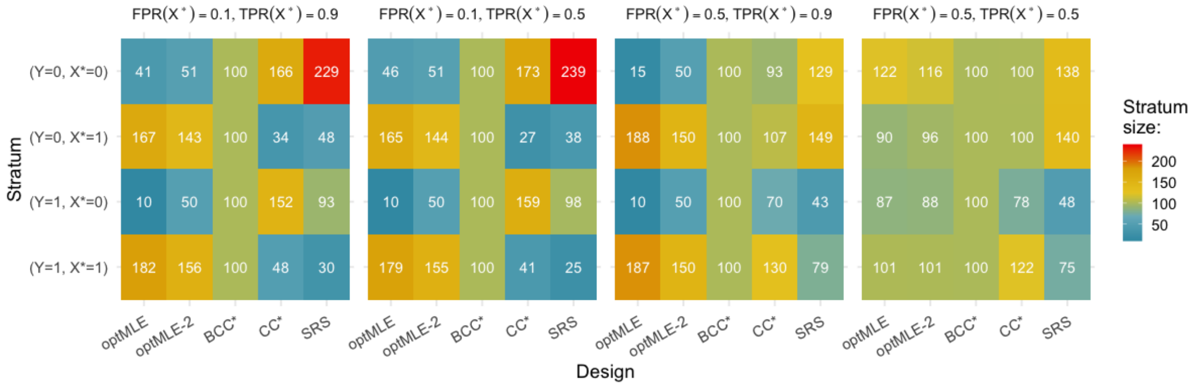

Figure S9. Average Phase II stratum sizes $\{n_{yx^*}\}$ under exposure misclassification.

Table S5. Historical TB audit results in CCASAnet. No subject had both outcome and exposure misclassification.

		**Misclassified (%)**	
**Country**	**Audited**	**Treatment Completion (Y^*)**	**Bacterial Confirmation (X^*)**
Country Grouping $Z = 0$			
A	6	2 (33.3%)	0 (0.0%)
B	7	0 (0.0%)	2 (28.6%)
Country Grouping $Z = 1$			
C	6	1 (16.7%)	1 (16.7%)
D	10	2 (20.0%)	5 (50.0%)
Country Grouping $Z = 2$			
E	4	0 (0.0%)	0 (0.0%)

Table S6. Parameter estimates for the TB analysis in CCASAnet using historical audits.

**Coefficient**	**log OR**
**Analysis model (Y)**	
Intercept	0.752
X	-0.415
$Z = 0$ (Country A–B)	Referent
$Z = 1$ (Country C–D)	0.601
$Z = 2$ (Country E)	0.211
**Outcome misclassification mechanism (Y^*)**	
Intercept	2.088
X^*	0.156
Y^*	4.644
X	2.485
$Z = 0$ (Country A–B)	Referent
$Z = 1$ (Country C–D)	-1.182
$Z = 2$ (Country E)	-0.956
**Exposure misclassification mechanism (X^*)**	
Intercept	-0.600
Y	-2.611
X	4.770
$Z = 0$ (Country A–B)	Referent
$Z = 1$ (Country C–D)	1.685
$Z = 2$ (Country E)	0.170
**Exposure model (X)**	
Intercept	-1.017
$Z = 0$ (Country A–B)	Referent
$Z = 1$ (Country C–D)	-0.160
$Z = 2$ (Country E)	-0.592

The optimal designs for CCASAnet were based on the historical parameters $\hat{\beta}^{(h)} = -0.415$ and $\hat{\boldsymbol{\eta}}^{(h)} = (0.752,\ 0,\ 0.601,\ 0.601,\ 0.211,\ 2.088,\ 0.156,\ 4.644,\ 2.485,\ 0,\ -1.182,\ -1.182,\ -0.956,\ -0.6,\ -2.611,\ 4.77,\ 0,\ 1.685,\ 1.685,\ 0.17,\ -1.017,\ 0,\ -0.16,\ -0.16,\ -0.592)^\top$.

Table S7. Simulation results comparing the MLE and SMLE.

	MLE				SMLE			
**Design**	**% Bias**	**SE**	**RE**	**RI**	**% Bias**	**SE**	**RE**	**RI**
optMLE	3.333	0.176	1.000	1.000	2.667	0.176	1.000	1.000
optMLE-2	1.000	0.181	0.942	0.933	0.333	0.181	0.946	0.934
BCC*	0.667	0.198	0.788	0.853	0.333	0.198	0.785	0.855
CC*	0.000	0.270	0.426	0.616	0.000	0.270	0.425	0.618
SRS	−5.667	0.305	0.333	0.541	−5.667	0.305	0.332	0.544

The SMLE was proposed with X^* as a surrogate for X such that $(Y \perp X^*) \mid X$. Thus, X^* was generated following a Bernoulli distribution with $\Pr(X^* = 1 \mid Y, X) = [1+\exp\{-(\gamma_0+\gamma_1 X)\}]^{-1}$. All other variables were generated as in Section 3.2, with $p_{y0} = 0.3$, $p_x = 0.1$, $\text{FPR}_{00}(Y^*) = 0.1$, $\text{TPR}_{00}(Y^*) = 0.9$, $\text{FPR}(X^*) = 0.1$, and $\text{TPR}(X^*) = 0.9$. The % Bias and SE are, respectively, the empirical percent bias and standard error of the estimators. Each entry is based on 1,000 replicates.
